# Hepatocyte growth factor, colony-stimulating factor 1, CD40, and 11 other inflammation-related proteins are associated with pain in diabetic neuropathy: exploration and replication serum data from the Pain in Neuropathy Study

**DOI:** 10.1097/j.pain.0000000000002451

**Published:** 2021-08-24

**Authors:** Emmanuel Bäckryd, Andreas Themistocleous, Anders Larsson, Torsten Gordh, Andrew S.C. Rice, Solomon Tesfaye, David L. Bennett, Björn Gerdle

**Affiliations:** a Pain and Rehabilitation Center, and Department of Health, Medicine and Caring Sciences, Linköping University, Linköping, Sweden; b Nuffield Department of Clinical Neurosciences, University of Oxford, Oxford, United Kingom; c Department of Medical Sciences, Clinical Chemistry, Uppsala University, Uppsala, Sweden; d Department of Surgical Sciences, Uppsala University, Uppsala, Sweden; e Pain Research, Departmennt Surgery and Cancer, Faculty of Medicine, Imperial College London, United Kingdom; f Diabetes Research Unit, Sheffield Teaching Hospitals NHS Foundation Trust, Sheffield, United Kingdom

**Keywords:** Biomarker, Chronic pain, Diabetes, Inflammation, Neuropathic, Neuropathy, Pain, Polyneuropathy

## Abstract

One in 5 patients with diabetes suffers from chronic pain with neuropathic characteristics, but the pathophysiological mechanisms underlying the development of neuropathic pain in patients with diabetic distal symmetrical polyneuropathy (DSP) are poorly understood. Systemic low-grade inflammation has been implicated, but there is still a considerable knowledge gap concerning its scope and meaning in this context. The aim of the study was to establish the broad inflammatory signature of painful diabetic DSP in serum samples from the Pain in Neuropathy Study, an observational cross-sectional multicentre study in which participants underwent deep phenotyping. In the present two cohorts exploration–replication study (180 participants in each cohort), serum samples from Pain in Neuropathy Study participants were analyzed with the Olink INFLAMMATION panel (Olink Bioscience, Uppsala, Sweden) that enables the simultaneous measurement of 92 inflammation-related proteins (mainly cytokines, chemokines, and growth factors). In both the exploration and the replication cohort, we identified a high-inflammation subgroup where 14 inflammation-related proteins in particular were associated with more neuropathy and higher pain intensity. The top 3 proteins were hepatocyte growth factor, colony-stimulating factor 1, and CD40 in both cohorts. In the exploratory cohort, additional clinical data were available, showing an association of inflammation with insomnia and self-reported psychological distress. Hence, this cross-sectional exploration–replication study seems to confirm that low-grade systemic inflammation is related to the severity of neuropathy and neuropathic pain in a subgroup of patients with diabetic DSP. The pathophysiological relevance of these proteins for the development of neuropathic pain in patients with diabetic DSP must be explored in more depth in future studies.

## Abstract 2

Supplemental Digital Content is Available in the Text. In a subgroup of patients with diabetic polyneuropathy, the severity of neuropathy and neuropathic pain were associated with high levels of systemic inflammatory biomarkers.

## 1. Introduction

A third to a half of patients with diabetes develop distal symmetrical polyneuropathy (DSP),^1,68,71^ and 25 to 50% of patients with diabetic DSP develop neuropathic pain. ^1,68^ One in 5 patients with diabetes suffers from chronic pain with neuropathic characteristics.^11^ Neuropathic pain is defined as pain arising as a consequence of a lesion or disease of the somatosensory nervous system^25^ and is very difficult to treat effectively.^24^

The pathophysiological mechanisms underlying the development of pain in patients with diabetic DSP are poorly understood, but the initial inciting event is a “dying back” axonopathy principally affecting sensory neurons, possibly involving a cutaneous immunogenic imbalance towards inflammation in which Langerhans cells may play a role.^23,64^ Risk factors for the development of painful DSP include obesity, glycemic burden, and female sex.^57^ Microvascular abnormalities of the vasa nervorum, hyperglycemia and its downstream effects (eg, advanced glycation end-products), and chronic low-grade inflammation are believed to be involved in injury and sensitization of peripheral neurons.^56,61^ These changes are not intrinsic to neurons, but alterations in Schwann cells and the nerve microenvironment are also likely to be contributory.^50,62^

Recent human biomarker studies in blood and cerebrospinal fluid indicate that different chronic pain conditions are associated with low-grade chronic inflammation and neuroinflammation. This has been shown for instance in chronic postsurgical neuropathic pain,^14^ trigeminal neuralgia,^21^ chronic widespread pain including fibromyalgia,^16,29,36^ and lumbar radicular pain.^48^ Immune cell profiles by flow cytometry can also be used to further ascertain the role of the immune system.^42^ Preclinical animal models of neural injury also link inflammation or neuroinflammation to the development of pain-related hypersensitivity.^31^

There is still a considerable knowledge gap concerning the scope and meaning of systemic low-grade inflammation in the development of painful DSP in humans. A prerequisite for the acquisition of such pathophysiological knowledge is the possibility to analyse not just a few candidate proteins (see, eg, Ludwig et al.^43^) but rather to simultaneously study an extensive network of cytokines, chemokines, and growth factors to get a more comprehensive image.

The aim of the study was to establish the broad inflammatory signature assessed in serum in the context of painful diabetic DSP. The goals of the project were to measure 92 inflammation-related proteins in serum collected from study participants, recruited as part of the Pain in Neuropathy Study (PiNS),^66^ with painful and painless diabetic DSP, and to relate the inflammation pattern to clinical data. The study contained an exploratory phase and a replication phase, with 180 participants in each phase.

## 2. Methods

### 2.1. Overview of procedures and rationale

In a first exploratory phase, serum samples from 180 PiNS patients were sent from Oxford to the biobank of Uppsala University Hospital, Sweden, and the samples were then analysed using a panel of 92 inflammation-related proteins as described below. The results were related to the extensive clinical data recorded in PiNS. Then, in a replication phase, serum samples of 180 new PiNS patients were handled in the same way.

An underlying assumption in the present work is that (partly) different pathophysiological mechanisms might be at work in subgroups of patients suffering from painful diabetic DSP. As expressed on a more general level by Comte et al., many clinical disease entities may be umbrella terms encompassing several “molecular diseases” that share prominent signs and symptoms.^17^ Concerning patients with DSP, chronic low-grade inflammation may be of greater importance for the development and maintenance of pain in some patients and less important in others. A simple comparison between painful and painless patients with DSP might therefore lead to the “drowning” of a putative inflammatory signal. Therefore, our main strategy in the present article was to first define a subgroup of patients with high inflammatory activity, the hypothesis being that such a subgroup would then be shown to be clinically meaningful. Our approach partly resembles that of Baron et al.,^5^ who clustered patients with peripheral neuropathic pain using quantitative sensory testing (QST), the difference being (1) that our clustering methodology was based on objective measures, ie, proteins and not based on a semiobjective psychophysical method such as QST, and (2) that we took the additional step of testing whether the high-inflammation subgroup made clinical sense using patient-report data (as described below). This approach is consistent with a system medicine perspective, in which groups of interest are defined using “mechanism-based stratification”^17^ instead of the more conventional focus on signs and symptoms.

### 2.2. Patients and clinical data

#### 2.2.1. General information about the Pain in Neuropathy Study

The Pain in Neuropathy Study is an observational cross-sectional multicentre study in which participants underwent deep phenotyping that included neuropathy screening tools, extensive symptom and function questionnaires, neurological examination, nerve conduction studies, QST, and skin biopsy for intraepidermal nerve fibre density (IENFD) assessment in a subset of patients.^66^ Patients with diabetes mellitus aged above 18 years with diagnosed DSP or patients with symptoms and signs suggestive of DSP were included. Exclusion criteria were pregnancy, coincident major psychiatric disorders, poor or no English language skills, severe pain at recruitment from a cause other than DSP (to prevent potential confounding influence on pain reporting as well as psychological and quality-of-life reported outcomes), patients with documented central nervous system lesions, or patients with insufficient mental capacity to provide informed consent or to complete questionnaires. Many of the study participants were recruited from primary care practices in London and Oxford. Study participants were also recruited from diabetes and other clinics at Chelsea and Westminster Hospital (London), Sheffield Teaching Hospitals and Oxford University Teaching Hospitals, neurology clinics at King's College Hospital (London), and through advertisements.

#### 2.2.2. Exploratory cohort

We analyzed the following clinical data on the 180 PiNS patients of the exploratory cohort:

1. Anthropometric data (weight [kg] and height [m])
2. Data pertaining to diabetes and metabolic control (body mass index [BMI; kg/m^2^], HbA1c, type of diabetes, and duration of diabetes)
3. Data related to neuropathy—the Toronto Clinical Scoring System (TCSS) correlates with diabetic neuropathy severity and was used as a screening tool for DSP^12^; intraepidermal nerve fiber density (IENFD) was also measured
4. Pain intensity measurements—Brief Pain Inventory (BPI) severity scores^65^; Numerical Rating Scale (NRS) for 7 days mean pain intensity by means of a pain diary
5. Questionnaires pertaining to psychological distress—the Depression Anxiety Positive Outlook instrument (DAPOS) was used to measure mood and anxiety^55^; the Pain Anxiety Symptom Scale 20 (PASS) to assess pain-related anxiety^47^
6. Insomnia—the Insomnia Severity Index (ISI).^6^

The methods and questionnaires have been previously described in detail.^66^

#### 2.2.3. Replication cohort

We analyzed 180 new patients in a replication cohort. This was collected as part of the DOLORisk project and was harmonized with the original PiNS study with some minor adjustments (for full DOLORisk protocol, see PMID: 30756091): Duration of diabetes, NRS 7 days mean pain intensity, DAPOS, PASS, IENFD, and ISI were not available in the replication cohort. Hence, the clinical part of the replication cohort was focused on neuropathy (TCSS), pain intensity data (BPI), anthropometric data, and glycaemic control. The exploratory and replications cohorts were similar for polyneuropathy and neuropathic pain gradings.

#### 2.2.4. Final analysis of clinical data of both cohorts together

After the replication phase of the study, clinical data from the 360 patients were analyzed together in a common final analysis, and at this stage, the following neuropathic pain questionnaires were included: the Douleur Neuropathique en 4 Questions (DN4),^9^ PainDETECT,^27^ and the Neuropathic Pain Symptom Inventory (NPSI) whereby neuropathic pain symptoms such as evoked pain, superficial spontaneous pain, deep spontaneous pain, paroxysmal pain, and paresthesia or dysesthesia were investigated.^10^ Moreover, the total score of the Pain Catastrophizing Scale was used to assess the cognitive process by which pain was appraised.^53^ Catastrophizing is characterized by a lack of confidence and control and an expectation of negative outcomes.

### 2.3. Inflammation data

#### 2.3.1. Blood samples

A 10 mL blood sample (BD Vacutainer SST Tubes) was drawn from each participant. After 30 minutes, to allow blood to clot, the sample was centrifuged at 3000 rpm for 10 minutes at a temperature of 4°C. Serum was then aliquoted into 1.8 mL Nunc CryoTubes and stored at −80°C.

#### 2.3.2. Proximity extension assay

First, 180 serum samples were analyzed with the Olink INFLAMMATION panel (Olink Bioscience, Uppsala, Sweden) in the exploratory phase of the study. Then, 180 new patient samples were analyzed in the replication phase. The Olink INFLAMMATION panel uses a proximity extension assay (PEA) technique that merges an antibody-based immunoassay with polymerase chain reaction (PCR) and quantitative real-time PCR (qPCR), enabling 92 inflammation-related protein biomarkers (mainly cytokines, chemokines, and growth factors) to be quantified simultaneously.^4,14,44^ Importantly, PEA is a dual-recognition immunoassay, where 2 matched antibodies labeled with unique DNA oligonucleotides simultaneously bind to a target protein. This brings the 2 antibodies into proximity, allowing their DNA oligonucleotides to hybridize; this is then followed by PCR.^51^ Hence, signal generation in PEA requires both dual-recognition and DNA sequence–specific protein-to-DNA conversion. The specifics of the PEA method have been explained elsewhere.^14^ A complete list of the 92 inflammation-related proteins, including their UniProt ID, is found in Supplemental Digital Content 1 (available at http://links.lww.com/PAIN/B474).

Olink INFLAMMATION panel data are expressed as normalized protein expression (NPX). Values of NPX are acquired by normalizing Cq values against extension control, as well as interplate control and a correction factor. They are on log2 scale. A high NPX value corresponds to a high protein concentration and can be linearized using the formula 2^NPX^. NPX can be used for statistical multivariate analysis and express relative quantification between samples but is not an absolute quantification.

### 2.4. Statistics

IBM SPSS Statistics (version 24.0; IBM Corporation, Route 100 Somers, New York, NY) was used for computations with the Kruskal–Wallis test, Mann–Whitney *U* test, χ^2^ test, and multiple linear regression (MLR) as appropriate. A significance level of 0.05 was chosen.

Details of multivariate data analysis (MVDA) methodology^22,69^ have been described in previous publications.^13–16,28,29,52^ In brief, we used SIMCA version 15 (Sartorius Stedim Biotech, Umeå, Sweden) for multivariate data analysis computations. We performed principal component analysis (PCA), hierarchical clustering analysis (HCA), and, based on the groups defined by HCA, orthogonal partial least squares–discriminant analysis (OPLS-DA). Principal component analysis is a technique that models the correlation structure of a data set and thereby enables the identification of multivariate outliers.^22,69^ Principal components extract relevant information found in the data, reducing a high-dimensional space (high number of variables) to a few “summary variables.” After outlier detection with PCA (strong outliers defined as Hotelling's T2»T2Crit(99%) and moderate outliers as DModX > 2*DCrit), we applied a bottom–up HCA to the principal component score vectors using the default Ward linkage criterion to identify relevant subgroups of patients. More precisely, we aimed at identifying a subgroup of patients characterized by high-inflammation activity, here defined as generally high levels of inflammation-related proteins in the abovementioned panel. Hierarchical clustering analysis complements PCA in the sense that while PCA identifies distinct clusters in multivariate space, HCA can find subtle clusters. In the resulting dendrogram, interesting patient subgroups were identified, and clinical data were compared between subgroups to ascertain the clinical relevance of the subgroups. Then, OPLS-DA was performed using group belonging as Y variables and protein data as predictors (X variables). To identify the proteins most relevant for group discrimination, the OPLS-DA models analyzed and identified associations between the X variables and group belonging. Multivariate data analysis analyzes all variables simultaneously, using the overall correlation pattern present in the data, hence separating information from “noise.” Hence, the protein data in this study were not primarily analyzed by multiple univariate testing, thereby minimizing the multiple testing problem.

### 2.5. Network analysis

The protein–protein association network for the important and common proteins for the cohorts were analysed using the online database tool Search Tool for the Retrieval of Interacting Genes/Proteins (STRING; version 11). Protein accession numbers (UniProt) for the most important and common significant proteins were entered in the search engine (multiple proteins) of STRING with the following parameters: organism: Homo sapiens, maximum number of interactions was query proteins only, interaction score was set to minimum required interaction score of medium confidence (0.400), a FDR ≤ 0.05 was used when classifying cellular component (CC), and molecular function and biological process (BP) according to the Gene Ontology (GO; http://geneontology.org/docs/ontology-documentation/) together with terms from Kyoto Encyclopedia of Genes and Genomes (KEGG) pathways obtained from STRING. Cellular component is the locations relative to cellular structures in which gene products perform function either cellular compartments or stable macromolecular complexes. Molecular function describes activities that occur at the molecular level and hence represents activities that can be performed by individual gene products, eg, a protein. Biological processes are larger processes or biological programs accomplished by multiple molecular activities; it is not equivalent to a pathway. In tables, we show the terms with FDR <0.001 or if a high number of terms with FDR <0.001, the 20 with lowest FDR. For the obtained network, protein–protein interaction (PPI) enrichment *P*-value and average local clustering coefficient were reported. In the network figure, each protein is represented by a coloured node, and PPI and association are represented by an edge visualized as a line. Higher combined confidence scores are represented by thicker lines/edges.

### 2.6. Ethics

The study was approved by the National Research Ethics Service of the United Kingdom (No.:10/H07056/35). All study participants signed written consent before participating.

## 3. Results

### 3.1. Exploration cohort

#### 3.1.1. Grouping of patients according to the overall inflammatory pattern

**Figure 1.**
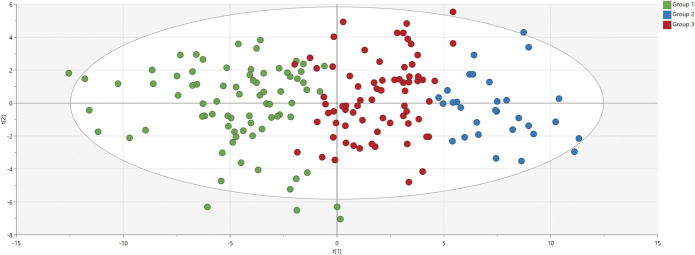
Principal component analysis score plot of the exploratory cohort. Each patient is a dot, and each dot is colored according to the hierarchical clustering analysis (HCA) group. Group 1 in green, Group 2 in blue, and Group 3 in red. The 2 axes t[1] and t[2] (scores) represent the 2 principle components of the model.

Eighteen inflammation-related proteins of 92 had >20% missing values and were therefore excluded from further analyses. The remaining 74 proteins were analyzed by PCA. One patient (ID 30160) was a strong outlier and was therefore excluded. The final PCA model (n = 179 and 74 proteins as X variables) had 4 PCs, R^2^ = 0.50, and Q^2^ = 0.38. Based on that, HCA was performed, and a level of 3 groups was chosen in the dendrogram (Supplemental Digital Content 2, available at http://links.lww.com/PAIN/B474). The 3 groups of patients are illustrated in the PCA scatter plot in Figure 1.

#### 3.1.2. Protein data in the 3 groups of patients

**Figure 2.**
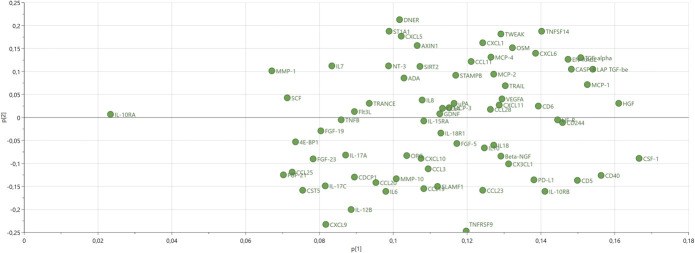
Principal component analysis loadings plot of the exploratory cohort. Each protein is a dot. The loadings plot is complementary to the score plot, and the loadings p[1] and p[2] “summarize” how the proteins relate to each other.

The 74 proteins of the PCA model are depicted in the corresponding PCA loading plot in Figure 2. The PCA score and loading plots being complementary to each other, Figure 1 and Figure 2 taken together show that Group 2 is a relative high-inflammation group, whereas Group 1 is characterized by the opposite, and Group 3 is intermediate. Extensive descriptive data tabulated in Supplemental Digital Content 3 (available at http://links.lww.com/PAIN/B474) confirmed this; and as can also be seen in the same supplement, omnibus testing (3 groups of patients) was highly statistically significant for all 74 proteins (ie, confirming the validity of the HCA).

#### 3.1.3. Clinical data in the 3 groups of patients

Statistically significant differences between the 3 groups were found for several of the clinical variables, that is, TCSS, several pain intensity variables, ISI, and PASS (Table 1). Based on descriptive data and omnibus tests shown in Table 1, we tested the hypothesis that Group 2 would differ from Group (1 + 3). Group 2 was significantly different with a generally more severe clinical situation than Group (1 + 3) in the following clinical variables: TCSS total score (*P* = 0.002), BPI pain severity subscore (*P* = 0.014), BPI pain average (*P* = 0.009), BPI worst (*P* = 0.020), BPI least (*P* = 0.040), BPI now (*P* = 0.015), NRS 7 days mean pain intensity (*P* = 0.015), ISI total score (*P* = 0.010), PASS cognitive (*P* = 0.007), PASS escape avoidance (*P* = 0.007), PASS physiological anxiety (*P* < 0.001), PASS fear (*P* = 0.014), PASS total (*P* = 0.003), DAPOS anxiety (*P* = 0.029), DAPOS depression (*P* = 0.022), and DAPOS positive outlook (*P* = 0.036). Age, gender, HbA1c, BMI, type of diabetes, duration of diabetes, and IENFD did not differ significantly between Group 2 and Group (1 + 3) (data not shown). In Group 2, 70% of patients had painful DSP, compared with 51% in Group (1 + 3) (*P* = 0.057).

**Table 1:** Exploratory cohort clinical data in 3 groups of patients.

Variable	Group 1 (n = 77)	Group 2 (n = 30)	Group 3 (n = 72)	*P*
Age (y)	68 (59-73)	69 (61-74)	65 (60-73)	0.619
Gender (% females)	30%	33%	26%	0.764
HbA1c (expressed in %)	7.3 (6.5-8.4)	7.3 (6.6-8.2)	7.8 (6.9-8.7)	0.199
Body mass index (BMI; kg/m^2^)	29.6 (26.4-35.9)	34.9 (26.6-38.1)	29.8 (26.8-33.2)	0.241
Duration of diabetes (y)	14 (8-23)	14 (10-21)	14 (9-21)	0.939
Type 2 diabetes	95%	83%	92%	0.157
TCSS total score	9 (6-11.5)	12 (9.5-16.5)	10 (7-13)	0.003*
NRS 7 days mean pain	0.7 (0-4.1)	3.8 (0-6.4)	0 (0-5)	0.037*
BPI pain severity subscore	1.4 (0-3.6)	3.8 (0-6)	0 (0-4.8)	0.048*
BPI pain average	1.5 (0-4.5)	4.5 (0-7)	0 (0-5.5)	0.031*
BPI worst	2 (0-6.5)	6 (0-8)	0 (0-7)	0.063
BPI least	0 (0-2)	1.5 (0-5)	0 (0-3)	0.121
BPI now	0 (0-3)	2.5 (0-7)	0 (0-4.5)	0.050*
IENFD	1.1 (0.4-1.8)	0.7 (0-1.4)	1.1 (0-1.9)	0.282
ISI total score	9 (3.5-14)	13 (8-22)	10 (4-17)	0.024*
PASS cognitive	5 (1-10)	9 (3.5-19.5)	5 (1.0-11.8)	0.026*
PASS escape avoidance	3 (0-9)	9 (3.3-15.8)	4 (2-12)	0.008*
PASS fear	2 (0-7)	6 (0.5-1.6)	1 (0-7)	0.047*
PASS physiological anxiety	0 (0-2)	4 (0-11)	0 (0-5)	0.002*
PASS total	12.5 (4-24.5)	31 (10-64)	10.5 (5-34)	0.011*
DAPOS anxiety	3 (3-6)	6 (3-8)	4 (3-6)	0.089
DAPOS depression	6 (5-10)	8 (5-16)	6 (5-10)	0.054
DAPOS positive outlook	12 (9-13)	9 (8-12.5)	11 (9-13)	0.099

The groups were determined by the pattern of inflammation-related proteins (cf. Fig. 1). Median (IQR) if not otherwise indicated. Statistics in the table is by the Kruskal–Wallis test. For comparisons of Group 2 vs Group (1 + 3) with the Mann–Whitney *U* test, see text.

*denotes statistical significance at the 0.05 level.

The BPI pain severity subscore is the mean of the 4 other reported BPI pain severity scores.

BPI, Brief Pain Inventory; DAPOS, Depression Anxiety Positive Outlook instrument; IENFD, intraepidermal nerve fiber density; ISI, Insomnia Severity Index; IQR, interquartile range; PASS, Pain Anxiety Symptom Scale 20; NRS, Numerical Rating Scale pain intensity 0 to 10; TCSS, Toronto Clinical Scoring System.

Hence, grouping of patients by HCA of protein levels was clinically meaningful; Group 2 being characterized by more neuropathy (ie, higher TCSS), higher pain intensity, more psychological distress, and increased insomnia compared with the 2 other groups.

#### 3.1.4. Cluster of proteins distinctive of Group 2

Next, we determined the proteins that were the most characteristic for Group 2. The PCA score and loading plots being complementary to each other, Figures 1 and 2 together show that Group 2 is particularly characterized by high levels of proteins having loadings > 0.13 (ie, p[1] values on the x-axis of Fig. 2). These proteins (and most notably colony-stimulating factor 1 (CSF-1), hepatocyte growth factor (HGF), and CD40, which had loadings > 0.15, see Fig. 2) were confirmed to be the most important ones by OPLS-DA comparing Group 2 with Group (1 + 3), with p(corr) values >0.66; the whole list of proteins can be seen in Supplemental Digital Content 4 (available at http://links.lww.com/PAIN/B474), in descending order of p(corr).

### 3.2. Replication cohort

#### 3.2.1. Grouping of patients according to the overall inflammatory pattern

**Figure 3.**
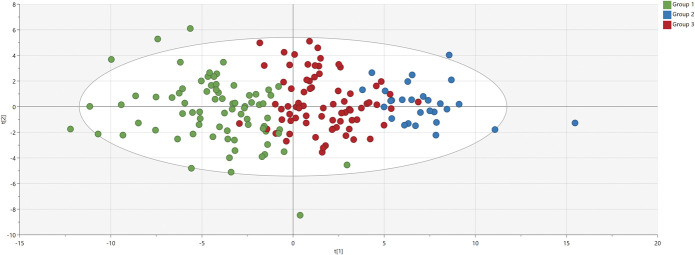
Principal component analysis score plot of the replication cohort. Each patient is a dot, and each dot is colored according to the hierarchical clustering analysis (HCA) group. Group 1 in green, Group 2 in blue, and Group 3 in red. The 2 axes t[1] and t[2] (scores) represent the 2 principle components of the model.

Nineteen inflammation-related proteins of 92 had >20% missing values and were therefore excluded from further analyses. The remaining 73 proteins were analyzed by PCA. One patient (ID 30419) was a strong outlier and was therefore excluded. The final PCA model (n = 179 and 73 proteins as X variables) had 4 PCs, R^2^ = 0.47, and Q^2^ = 0.34. Based on that, HCA was performed and a level of 3 groups could be replicated in the dendrogram (Supplemental Digital Content 2, available at http://links.lww.com/PAIN/B474). The 3 groups of patients were of similar size compared with the exploratory cohort and are illustrated in the PCA scatter plot in Figure 3. Hence, we were able to replicate 3 similar groups of patients, called groups 1 to 3 (as per the exploratory cohort).

#### 3.2.2. Protein data in the 3 groups of patients

**Figure 4.**
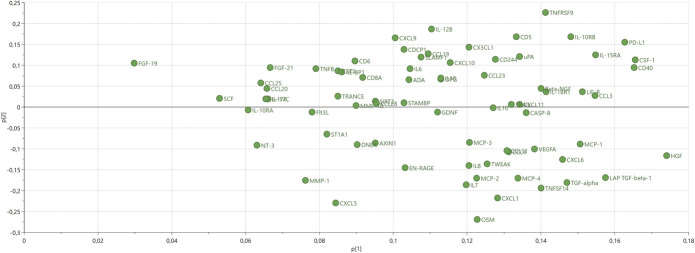
Principal component analysis loadings plot of the replication cohort. Each protein is a dot. The loadings plot is complementary to the score plot, and the loadings p[1] and p[2] “summarize” how the proteins relate to each other.

The 73 proteins of the PCA model are depicted in the corresponding PCA loading plot in Figure 4. The PCA score and loading plots being complementary to each other, Figures 3 and 4 together, show that Group 2 is a relative high-level-of-protein group, whereas Group 1 is characterized by the opposite, and Group 3 is intermediate. This interpretation was confirmed by descriptive analyses, and data are presented in Supplemental Digital Content 5 (available at http://links.lww.com/PAIN/B474). In addition, omnibus testing (3 groups of patients) was highly statistically significant for all 73 proteins (confirming the validity of the HCA). Hence, findings from the exploratory cohort were replicated.

#### 3.2.3. Clinical data in the 3 groups of patients

As shown in Table 2, we were able to replicate findings about Group 2 being characterized by more neuropathy and higher pain intensities for “BPI pain severity subscore,” “BPI pain average,” and “BPI worst,” essentially confirming the findings of the exploratory cohort (Table 1). The same questionnaires measuring psychological distress and insomnia were not available in the replication cohort. Comparing Group 2 with Group (1 + 3), we found that TCSS total score and BPI pain average were significantly higher in Group 2 (*P* = 0.022 and *P* = 0.048, respectively).

**Table 2:** Replication cohort clinical data in 3 groups of patients.

Variable	Group 1 (n = 76)	Group 2 (n = 28)	Group 3 (n = 75)	*P*
Age (y)	71 (63-76)	74 (65-78)	72 (66-78)	0.360
Gender (% females)	28%	46%	24%	0.078
HbA1c (expressed in %)	7.3	7.9	7.4	0.371
Body mass index (BMI; kg/m^2^)	27.9 (25.7-32.3)	30.1 (26.3-35.9)	29.0 (26.6-33.0)	0.239
Type 2 diabetes (%)	88%	93%	95%	0.341
TCSS total score	10 (6.8-12)	13 (10-16)	11 (8-14)	0.006*
BPI pain severity subscore	0 (0-4.3)	3.9 (0-6.4)	3.3 (0-5.6)	0.043*
BPI pain average	0 (0-4)	5 (0-6)	3 (0-6)	0.024*
BPI worst	0 (0-6)	5.5 (0-8)	4 (0-8)	0.037*
BPI least	0 (0-2)	1.5 (0-5)	0 (0-3)	0.128
BPI now	0 (0-3.3)	2 (0-5.8)	0 (0-6)	0.109

BPI, Brief Pain Inventory (the BPI Pain severity subscore is the mean of the 4 other reported BPI pain severity scores); BMI, body mass index; TCSS, Toronto Clinical Scoring System.

Numerical Rating Scale 7 days mean pain, Depression Anxiety Positive Outlook instrument (DAPOS), Pain Anxiety Symptom Scale 20 (PASS), intraepidermal nerve fibre density (IEFND), and Insomnia Severity Index (ISI) were not available in the replication cohort.

The groups were determined by the pattern of inflammation-related proteins. Median (IQR) if not otherwise indicated. Statistics in the table is by the Kruskal–Wallis test. For comparisons of Group 2 vs Group (1 + 3) with the Mann–Whitney *U* test, see text.

*denotes statistical significance at the 0.05 level.

#### 3.2.4. Cluster of proteins distinctive of Group 2

Next, we determined the proteins that were most characteristic for Group 2. The PCA score and loading plots being complementary to each other, Figures 3 and 4 together, show that Group 2 is particularly characterized by relatively high levels of proteins having loadings > 0.13 (ie, p[1] values on the x-axis of Fig. 4). These proteins were confirmed to be the most important ones by an OPLS-DA analysis comparing Group 2 to Group (1 + 3), with p(corr) values >0.62; the whole list of proteins can be seen in Supplemental Digital Content 4 (available at http://links.lww.com/PAIN/B474).

### 3.3. Comparison of the protein results of the 2 cohorts

The top 20 proteins by OPLS-DA from both cohorts are listed in Table 3; we found that 14 of the top 20 proteins in the exploratory cohort (ie, 70%) were among the top 20 proteins of the replication cohort. Hence, the following 14 proteins were replicated as being distinctive of Group 2: HGF, CSF-1, CD40, PD-L1, LAPTGF-beta-1, LIF-R, IL-10RB, monocyte chemotactic protein 1, TGF-alpha, CXCL6, beta-nerve growth factor, TNFSF14, CD5, and CASP-8. Moreover, the top 3 proteins are the same in both cohorts: HGF, CSF-1, and CD40 (Table 3). The whole list of proteins, in descending order of p(corr), for both cohorts, can be viewed in Supplemental Digital Content 4 (available at http://links.lww.com/PAIN/B474).

**Table 3:** Inflammation-related proteins characteristic of Group 2 for the exploratory cohort (left) and the replication cohort (right).

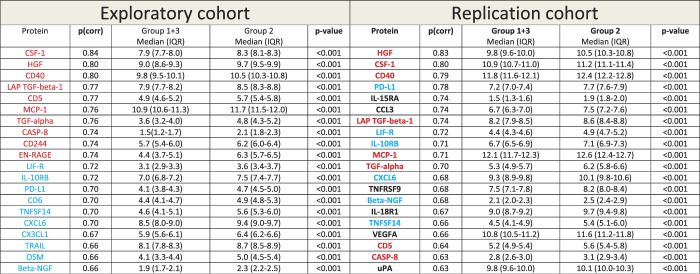

Note: IQR, interquartile range. To illustrate the overlap in results between the 2 OPLS-DA models, the top 10 proteins of the exploratory cohort are marked in red in both columns of the table. Likewise, the top 11 to 20 proteins of the exploratory cohort are marked in blue in both columns. Hence, 70% of the top 20 proteins in the exploratory cohort are among the top 20 proteins of the replication cohort. Moreover, the top 3 proteins are the same in both cohorts.

The top 20 proteins are listed in falling order of p(corr), that is, in falling order of importance for group discrimination by OPLS-DA (Group 2 vs Group [1 + 3]). Protein levels (median [IQR]) are shown in normalized protein expression (NPX), which is a relative quantification on a log2 scale (see Methods section); Group 2 is compared with Group 1 + 3.

MCP-1, monocyte chemotactic protein 1; NGF, nerve growth factor; OPLS-DA, orthogonal partial least squares–discriminant analysis.

### 3.4. Network analysis

**Figure 5.**
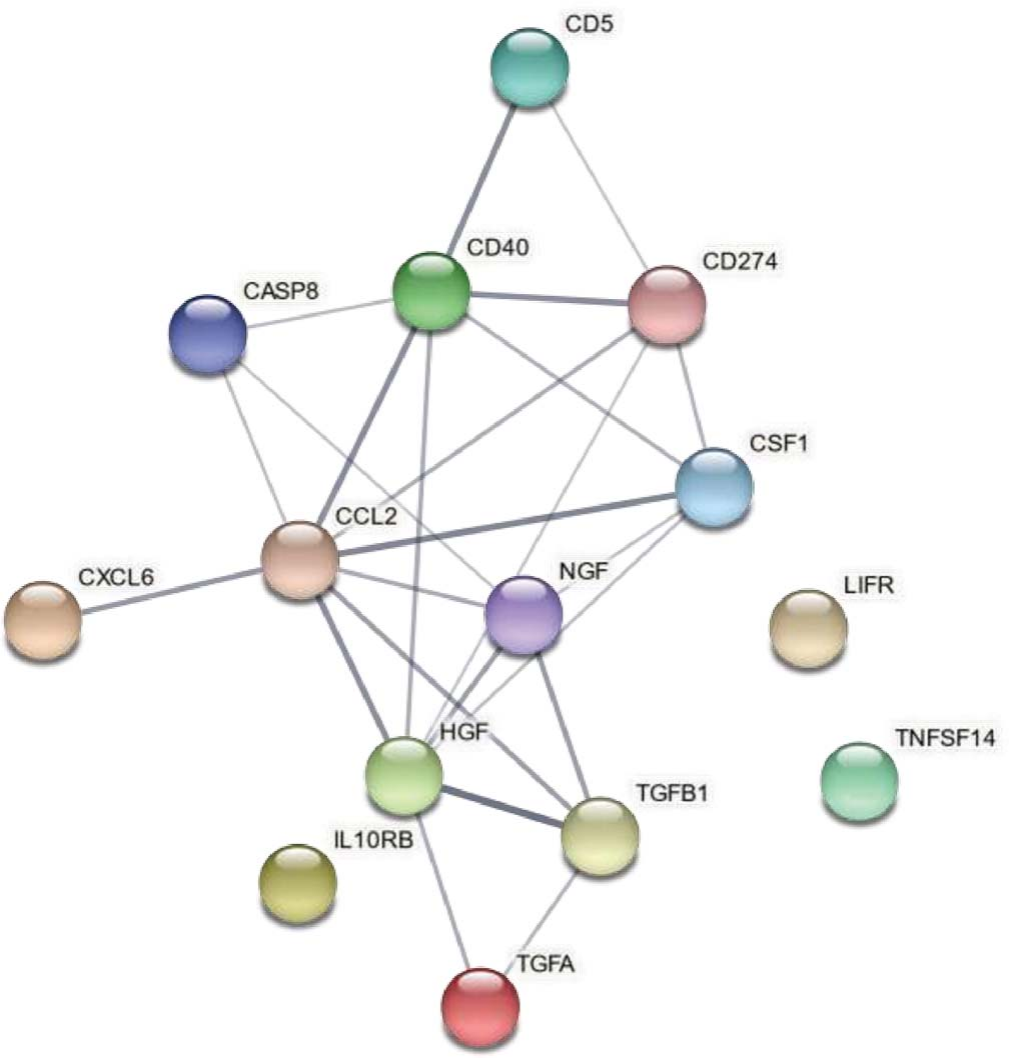
Network based on the 14 proteins common for the 2 cohorts. It had the following characteristics: number of nodes: 14, number of edges: 24, average node degree: 3.43, average local clustering coefficient: 0.566, expected number of edges: 4, protein–protein interaction (PPI) enrichment *P*-value: 3.26e-11.

The network and enrichment analysis of the 14 proteins (common to the exploration and replication cohorts) based on the STRING database identified a PPI network that was highly and significantly enriched (PPI enrichment *P*-value: 3.26e-11) (Fig. 5). Hence, most of these proteins are known to interact.

We have identified totally 85 GO terms with FDR values <0.001: molecular function (n = 7), CC (n = 2), and BP (n = 76). The majority concerned the BP. Cellular component of GO function enrichment analysis of the proteins identified 2 significant terms—both concerning extracellular compartment aspects (Table 4). According to the molecular function of GO, several terms related to receptor binding or activity as well as cytokine and growth factor activity had low FDR for the included proteins (Table 4). Among the 20 terms of the BP of GO with lowest FDR were responses, pathways and receptor binding relating to cytokines, regulation of the immune system, but also regulation of intracellular signal transduction and regulation of cells (Table 4). The KEGG pathway with lowest FDR was cytokine–cytokine receptor interaction (Table 4). In addition, terms relating to the Ras-Raf-MEK-ERK pathway, IL-17 signalling, TNF signalling, and PI3K-Akt signalling pathways were significant (Table 4). A complete list of the significant GO terms and KEGG pathways is shown in Supplemental Digital Content 6 (available at http://links.lww.com/PAIN/B492).

**Table 4:** The most significant Gene Ontology terms within cellular component, molecular function, and biological process together with the most enriched Kyoto Encyclopedia of Genes and Genomes pathways.

Category	#Term ID	Term description	Gene count	Strength	FDR	Matching proteins in the network
CC	GO:0005576	Extracellular region	11	0.79	4.26e-06	TGFB1, HGF, CCL2, CXCL6, LIFR, TGFA, CSF1, NGF, CD40, CD274, and TNFSF14
CC	GO:0005615	Extracellular space	8	0.99	1.57e-05	TGFB1, HGF, CCL2, CXCL6, TGFA, CSF1, CD274, and TNFSF14
MF	GO:0005126	Cytokine receptor binding	8	1.61	4.55e-10	TGFB1, CCL2, CXCL6, LIFR, CSF1, CASP8, NGF, and TNFSF14
MF	GO:0048018	Receptor ligand activity	8	1.39	1.32e-08	TGFB1, HGF, CCL2, CXCL6, TGFA, CSF1, NGF, and TNFSF14
MF	GO:0005102	Signaling receptor binding	10	0.97	1.52e-07	TGFB1, HGF, CCL2, CXCL6, LIFR, TGFA, CSF1, CASP8, NGF, and TNFSF14
MF	GO:0008083	Growth factor activity	5	1.64	1.55e-06	TGFB1, HGF, TGFA, CSF1, and NGF
MF	GO:0005125	Cytokine activity	5	1.51	5.54e-06	TGFB1, CCL2, CXCL6, CSF1, and TNFSF14
MF	GO:0032813	Tumor necrosis factor receptor superfamily binding	3	1.96	7.74e-05	CASP8, NGF, and TNFSF14
MF	GO:0005123	Death receptor binding	2	2.19	0.0010	CASP8 and NGF
BP	GO:0034097	Response to cytokine	11	1.17	4.74e-09	TGFB1, HGF, CCL2, CXCL6, LIFR, IL10RB, CSF1, CASP8, CD40, CD274, and TNFSF14
BP	GO:0019221	Cytokine-mediated signaling pathway	9	1.28	7.60e-08	TGFB1, HGF, CCL2, CXCL6, LIFR, IL10RB, CSF1, CD40, and TNFSF14
BP	GO:0007166	Cell surface receptor signaling pathway	12	0.88	1.59e-07	TGFB1, HGF, CCL2, CXCL6, LIFR, IL10RB, CSF1, CASP8, NGF, CD40, CD274, and TNFSF14
BP	GO:0002684	Positive regulation of the immune system process	9	1.15	5.15e-07	TGFB1, CCL2, CXCL6, CSF1, CD5, CASP8, CD40, CD274, and TNFSF14
BP	GO:0010469	Regulation of the signaling receptor activity	8	1.29	5.15e-07	TGFB1, HGF, CCL2, CXCL6, TGFA, CSF1, NGF, and TNFSF14
BP	GO:1902533	Positive regulation of intracellular signal transduction	9	1.12	6.75e-07	TGFB1, HGF, CCL2, TGFA, CSF1, CASP8, NGF, CD40, and TNFSF14
BP	GO:0048584	Positive regulation of response to stimulus	11	0.87	9.25e-07	TGFB1, HGF, CCL2, CXCL6, TGFA, CSF1, CASP8, NGF, CD40, CD274, and TNFSF14
BP	GO:0010033	Response to the organic substance	12	0.78	9.61e-07	TGFB1, HGF, CCL2, CXCL6, LIFR, IL10RB, CSF1, CASP8, NGF, CD40, CD274, TNFSF14
BP	GO:0030334	Regulation of cell migration	8	1.17	1.53e-06	TGFB1, HGF, CCL2, CXCL6, CSF1, CD40, CD274, and TNFSF14
BP	GO:0030335	Positive regulation of cell migration	7	1.34	1.53e-06	TGFB1, HGF, CXCL6, CSF1, CD40, CD274, and TNFSF14
BP	GO:0042127	Regulation of cell population proliferation	10	0.94	1.53e-06	TGFB1, HGF, CCL2, CXCL6, LIFR, TGFA, CSF1, NGF, CD40, and CD274
BP	GO:0071310	Cellular response to organic substance	11	0.84	1.53e-06	TGFB1, HGF, CCL2, CXCL6, LIFR, IL10RB, CSF1, CASP8, NGF, CD40, and TNFSF14
BP	GO:0051251	Positive regulation of the lymphocyte activation	6	1.51	1.71e-06	TGFB1, CCL2, CD5, CD40, CD274, and TNFSF14
BP	GO:0007165	Signal transduction	13	0.58	6.81e-06	TGFB1, HGF, CCL2, CXCL6, LIFR, IL10RB, CSF1, CD5, CASP8, NGF, CD40, CD274, and TNFSF14
BP	GO:0002685	Regulation of leukocyte migration	5	1.6	7.03e-06	TGFB1, CCL2, CXCL6, CSF1, and TNFSF14
BP	GO:0045785	Positive regulation of cell adhesion	6	1.35	7.71e-06	TGFB1, CCL2, CSF1, CD5, CD274, and TNFSF14
BP	GO:0008283	Cell population proliferation	7	1.16	8.92e-06	TGFB1, HGF, TGFA, CSF1, CD5, CD40, and TNFSF14
BP	GO:0048522	Positive regulation of the cellular process	13	0.57	8.92e-06	TGFB1, HGF, CCL2, CXCL6, LIFR, TGFA, CSF1, CD5, CASP8, NGF, CD40, CD274, and TNFSF14
BP	GO:0050920	Regulation of chemotaxis	5	1.57	8.92e-06	TGFB1, CCL2, CXCL6, CSF1, and TNFSF14
BP	GO:0050870	Positive regulation of T-cell activation	5	1.56	9.18e-06	TGFB1, CCL2, CD5, CD274, and TNFSF14
KEGG	hsa04060	Cytokine–cytokine receptor interaction	9	1.68	2.54e-12	TGFB1, HGF, CCL2, CXCL6, LIFR, IL10RB, CSF1, CD40, and TNFSF14
KEGG	hsa05144	Malaria	4	2.08	1.60e-06	TGFB1, HGF, CCL2, and CD40
KEGG	hsa05323	Rheumatoid arthritis	4	1.82	9.82e-06	TGFB1, CCL2, CXCL6, and CSF1
KEGG	hsa05145	Toxoplasmosis	4	1.71	2.01e-05	TGFB1, IL10RB, CASP8, and CD40
KEGG	hsa04010	MAPK signaling pathway	5	1.38	2.26e-05	TGFB1, HGF, TGFA, CSF1, and NGF
KEGG	hsa04014	Ras signaling pathway	4	1.39	0.00022	HGF, TGFA, CSF1, and NGF
KEGG	hsa05211	Renal cell carcinoma	3	1.79	0.00022	TGFB1, HGF, and TGFA
KEGG	hsa04657	IL-17 signaling pathway	3	1.66	0.00039	CCL2, CXCL6, and CASP8
KEGG	hsa05142	Chagas disease (American trypanosomiasis)	3	1.62	0.00045	TGFB1, CCL2, and CASP8
KEGG	hsa04668	TNF signaling pathway	3	1.59	0.00049	CCL2, CSF1, and CASP8
KEGG	hsa04151	PI3K-Akt signaling pathway	4	1.21	0.00065	HGF, TGFA, CSF1, and NGF

For explanation of proteins, see Supplemental Digital Content 1 (see http://links.lww.com/PAIN/B474). Note that for BP is shown the 20 terms with lowest FDR.

BP, biological process; CSF, colony-stimulating factor 1; CC, cellular component; GO, Gene Ontology; Gene count, observed gene count; HGF, hepatocyte growth factor; FDR, false discovery rate; KEGG, Kyoto Encyclopedia of Genes and Genomes. MF, molecular function; NGF, nerve growth factor.

### 3.5. Clinical data of both cohorts together

We then analyzed clinical data in both cohorts together, now also including data from neuropathic pain questionnaires (DN4, NPSI, and PainDetect) as well as the Pain Catastrophizing Scale. Descriptive data in the boxplot format can be viewed in Supplemental Digital Content 7 (available at http://links.lww.com/PAIN/B474). Comparing Group 2 with Group (1 + 3) for clinical data, we found that Group 2 had significantly higher values for TCSS (*P* < 0.001), BPI pain average (*P* = 0.001), BPI severity subscore (*P* = 0.003), BPI worst (*P* = 0.004), BPI least (*P* = 0.013), BPI now (*P* = 0.014), DN4 (*P* = 0.001), PainDetect (*P* = 0.020), NPSI superficial spontaneous pain (*P* = 0.015), NPSI paroxysmal pain (*P* = 0.007), NPSI paresthesia/dysesthesia (*P* = 0.003), NPSI total (*P* = 0.03), and BMI (*P* = 0.044).

### 3.6. Regression of DN4

Because, as shown above in section 3.5, BMI was significantly higher in group 2 vs (1 + 3), we used MLR to adjust for BMI by regressing DN4 (the dependent variable) with 2 predictors (ie, independent variables), namely, BMI and the first PC of a PCA model of the 14 proteins as per section 3.3 (henceforward called PC1_14prot). Hence, PC1_14prot was used as a “summary” variable for the 14 proteins. This was performed first in the exploration cohort, and then, the same analysis was performed in the replication cohort. We chose DN4 as the dependent variable because it was the most normally distributed outcome variable available (ie, not skewed).

#### 3.6.1. Regression of Douleur Neuropathique en 4 Questions in the exploration cohort

In the PCA model, PC1_14prot captured 58% of the variation. The MLR model was significant (adjusted R^2^ = 0.041; *P* = 0.010), and PC1_14prot was significantly and positively associated with DN4 (significant coefficient, *P* = 0.01), when adjusting for BMI.

#### 3.6.2. Regression of Douleur Neuropathique en 4 Questions in the replication cohort

In the PCA model, PC1_14prot captured 55% of the variation. The MLR model was significant (adjusted R^2^ = 0.097; *P* <0.001), and PC1_14prot was significantly and positively associated with DN4 (significant coefficient, *P* = 0.044), when adjusting for BMI.

### 3.7. Summary

In this exploration–replication study of patients with DSP, we identified a cluster of 14 inflammation-related proteins associated with more neuropathy, higher pain intensity, more severe neuropathic pain (NPSI), and higher scores for DN4 and PainDETECT (higher scores represent a higher likelihood of neuropathic pain). The top 3 proteins were HGF, CSF-1, and CD40 in both cohorts. In the exploratory cohort, additional clinical data were available, also showing an association with insomnia and self-reported psychological distress (PASS). Finally, as a contrast to the main methodology used in this article, the results of a more conventional approach are reported in Supplemental Digital Content 8 (available at http://links.lww.com/PAIN/B474), that is, participants with painful DSP are compared with participants with painless DSP (focusing on the exploratory cohort). Although the models reported in Supplemental Digital Content 8 (available at http://links.lww.com/PAIN/B474) are not statistically significant, the top 20 proteins responsible for group discrimination (painful vs painless DSP) overlap to a high degree (80%) with the 20 proteins listed in Table 3 above.

## 4. Discussion

The “translational gap” between animal experiments and clinical studies remains a challenge in pain research.^2,46^ Against that background, biomarker studies on people living with pain can be viewed as an important endeavour, the aim being to contribute to a better understanding of the pathophysiological mechanisms involved. Compared with other human biomarker studies in the field of chronic pain in general and of painful DSP in particular, this study has 3 major strengths.

First, our study included a replication phase, whereby many of the findings in the first cohort were confirmed in the second. In the context of a purported reproducibility crisis,^7,8,26,34,35,45,54^ this is arguably no small advantage. Not only were the patient groups defined by HCA remarkably replicable (c.f. the dendrograms in Supplemental Digital Content 2 (available at http://links.lww.com/PAIN/B474) and the substantial overlap of proteins results, see Table 3), in both the exploratory and the replication cohorts, but we also found that the HCA grouping was clinically relevant.

Second, we did not examine only a few candidate biomarkers of low-grade inflammation. Instead, we used a well-established and commercially available panel enabling the analysis of up to 92 inflammation-related proteins at the same time. As proteins work within networks, such a multiplexed approach does make sense in the search for a better understanding of biological and pathophysiological processes.^39,51^

Third, we used an unbiased clustering approach based on serum protein levels rather than on the basis of patient-reported outcome measures ^15^ or psychophysical tests such as sensory phenotyping,^5^ which are important but will be influenced by the multiplicity of psychological and social factors that can affect pain reports.^19,67^ This approach is methodologically advantageous when investigating a particular pathophysiological mechanism. Hence, we shifted perspective by letting biology define the groups of interest. Not only that, but because we let the correlation structure of proteins define the groups, one could say that we let systems biology^3^ define the 3 groups. We then used patient-reported outcome measures to see it the groups defined by biology made sense clinically—which they did. In addition, as stated above, the findings were replicable.

It could be argued that there is a big overlap in clinical data between the 3 groups of patients. That is, true; for instance, some patients in Group 1 reported significant pain, whereas some patients in Group 2 reported no pain. However, that is to be expected. We of course do not contend that the putative pathophysiological processes, that our study might be mirroring, are the only mechanisms underlying the development and maintenance of neuropathic pain in DSP. On the contrary, it is reasonable to believe that the biological mechanisms are much more complex and that our study mirror one part of that complexity. Although the presented OPLS-DA regressions (group 2 vs Group [1 + 3]) were highly significant, they only explained half (45%-46%) of the variation in group membership (Supplemental Digital Content 4). Given the fact that we were able to replicate our findings, we do however think that it is reasonable to conclude that our results mirror a biological reality. This also confirmed by the fact that the additional models presented in Supplemental Digital Content 8 (available at http://links.lww.com/PAIN/B474), although not statistically significant, largely confirmed the main findings summarized in Table 3.

An obvious limitation is the fact that this study had a cross-sectional design, making the assessment of causality a challenge. In addition, the possibility of confounders must be acknowledged. For instance, medication has not been taken into account. In addition, although BMI did not differ significantly between the 3 groups when looking at the 2 cohorts separately (Tables 1 and 2), there was nonetheless a trend for higher BMI in Group 2. In addition, when clinical data from both cohorts were analyzed together (section 3.5), patients in Group 2 had a higher BMI (*P* = 0.044, probably reflecting higher statistical power). This is important when considering that obesity or the metabolic syndrome is known to be associated with systemic low-grade inflammation^18,33,49,59^; do our results merely mirror that fact? It is of course a possibility, but one could also in that case argue for a causal relationship going from obesity to systemic inflammation, the latter then playing a role as a risk factor for the development of painful DSP. Whether the low-grade systemic inflammation associated with painful DSP “originates” in adipose tissue, or locally in the immediate vicinity of the injured nerves, or both, or somewhere else, cannot be answered by this study. However, the regression of DN4 with BMI and the first PC of the 14 proteins (a “summary measure” of these proteins) as predictors showed that the PC was a significant predictor even when adjusting for BMI. Given what DN4 measures, one could perhaps say that it seems that inflammation (here defined by PC1_14prot) is associated with a higher likelihood that pain in these patients with DSP is neuropathic in nature, even when adjusting for BMI. Hence, our findings should not be simplistically discarded as resulting from a confounding effect of obesity.

Some of the main protein findings will now be highlighted. Among the classical inflammation-related proteins often cited in the context of painful DSP,^56,57,61^ our study has confirmed that nerve growth factor and monocyte chemotactic protein 1 (also known as CCL2) both belong to the cluster of 14 proteins overrepresented in group 2 that has a high prevalence of neuropathic pain. We also identified several other interesting proteins, of whom the top 3 will now be briefly discussed.

The expression of HGF is increased in injured peripheral nerves, and HGF seems to play a role in Schwann cell–mediated nerve repair.^40^ Hence, the high levels in Group 2 would be indicative of more nerve damage. Hepatocyte growth factor gene therapy has been proposed as a possible treatment for diabetic neuropathy.^38^ Macrophage CSF-1^70^ is a cytokine which, in mouse models of Charcot–Marie–Tooth neuropathy, plays a role in macrophage-mediated neural damage.^32^ In addition, CD40L receptor (CD40), a member of the TNF-receptor superfamily, has an increased expression in diabetic nerves and seems to be a key molecule for the upregulation of hypoxia-inducible factor-1α (HIF-1α).^37^ A relationship between HIF-1α (a regulator of oxygen homeostasis) and pain intensity in DSP patients has been reported.^62^ See also Supplemental Digital Content 9 (available at http://links.lww.com/PAIN/B474). All in all, a coherent image emerges, whereby these and other inflammation-related proteins can reasonably be hypothesized to play a role in the development of neuropathic pain in DSP.

Some of the proteins in Table 3 have also been major findings in other human biomarker studies in the chronic pain field. In patients with chronic widespread pain or fibromyalgia, high levels of LAP TGF-beta-1,^16,29^ CASP-8,^29^ CXCL6,^16^ and HGF^29^ were found in blood. These results, albeit from nociplastic pain conditions, nevertheless confirm the pain relevance of our findings.

The expression of cytokines by peripheral blood mononuclear cells has been shown to be increased in patients with peripheral neuropathy, and proinflammatory cytokines and growth factors have been implicated in the pathogenesis of neuropathic pain.^20,41,63^ It is worth bearing in mind that the dichotomy between proinflammatory and anti-inflammatory cytokines is by no means self-evident and absolute. For instance, IL-10 (often considered the most important anti-inflammatory cytokine in humans^60^) can have proinflammatory properties in some circumstances.^30,60^ Moreover, it seems sensible to postulate that also the production of anti-inflammatory cytokines could be elevated in a high-inflammation group associated with chronic pain, as part of an (insufficient) compensatory mechanism.^58^ The network analysis is briefly discussed in Supplemental Digital Content 10 (available at http://links.lww.com/PAIN/B474).

Using the same panel as in this study, Ziegler et al. compared 3 groups: diabetics with DSP, diabetics without DSP, and persons with normal glucose tolerance without polyneuropathy.^72^ The authors found that 17 inflammation-related proteins were lower in the DSP group. However, and importantly, the cohorts came from different studies; only univariate testing was effectuated (ie, unlike in this study, the correlation structure of the whole material was not taken into account); the focus of the study was neuropathy, not neuropathic pain. Therefore, although we used the same panel, our 2 studies are difficult to compare. Still, in an additional analysis very briefly described in the article, Ziegler et al. did compare painful DSP with painless DSP with a conservative Bonferroni correction to account for multiple univariate testing (ie, increasing the risk for type 2 errors); no differences were detected between the groups, and data were simply “not shown.”

### 4.1. Conclusion

The abovementioned limitations notwithstanding this exploration–replication study seems to confirm that low-grade systemic inflammation is related to the severity of neuropathy and neuropathic pain in a subgroup of patients with DSP. Several interesting inflammation-related proteins were identified including, but not limited to, previously described findings. Hepatocyte growth factor, CSF-1, and CD40 were particularly highlighted in both cohorts. The pathophysiological relevance of these proteins for the development of neuropathic pain in patients with DSP must be explored in more depth in future studies.

## Conflict of interest

D.L. Bennett has acted as a consultant in the last 2 years for Amgen (2020), Bristows (2020), LatigoBio (2021), GSK (2021), Lilly (2020), Mundipharma, Olipass (2020), Orion, Regeneron (2020), and Theranexus (2020) on behalf of Oxford University Innovation. B. Gerdle is currently (since 2020) involved in a collaboration with Pfizer Inc. concerning chronic low back pain and osteoarthritis. A.S.C. Rice declares the interests occurring in the past 24 months: (1) Consultancy and advisory board work for Imperial College Consultants; in the past 24 months, this has included remunerated work for Abide, Confo, Vertex, Pharmanovo, Lateral, Novartis, Mundipharma, Orion, Shanghai SIMR Biotech, Asahi Kasei, Toray, and Theranexis. (2) Owner of share options in Spinifex Pharmaceuticals from which personal benefit accrued on the acquisition of Spinifex by Novartis in July 2015 and from which milestone payments were made until 2019. (3) Inventor on patents WO 2005/079771 & EP13702262.0/WO2013 110945. Neither being actively pursued at the current time. (4) Member, Joint Committee on Vaccination and Immunisation, Varicella subcommittee. (5) Member, Neurology, Pain & Psychiatry Expert Advisory Group, Commission on Human Medicines, Medicines & Healthcare Products Regulatory Agency (MHRA). S. Tesfaye declares potential dualities of interests over the past 5 years. Over the past 5 years, he has received honoraria as speaker fee from Novo Nordisk, Pfizer, Merk, Eva Pharma, Hikma, Grunenthal, Astellas Pharma, Abbott, and AstraZeneca. He has also been or is on the Advisory Board of Nevro, Bayer, Trigocare International, Worwag Pharma, Angelini, and Mitsubishi Tanabe Pharma. The remaining authors have no conflicts of interest to declare.

## Appendix A. Supplemental digital content

Supplemental digital content associated with this article can be found online at http://links.lww.com/PAIN/B474 and http://links.lww.com/PAIN/B492

## References

[1] Abbott CA Malik RA van Ross ER Kulkarni J Boulton AJ. Prevalence and characteristics of painful diabetic neuropathy in a large community-based diabetic population in the U.K. Diabetes Care 2011;34:2220–4. 21852677 10.2337/dc11-1108 PMC3177727

[2] Andrews NA Latrémolière A Basbaum AI Mogil JS Porreca F Rice ASC Woolf CJ Currie GL Dworkin RH Eisenach JC Evans S Gewandter JS Gover TD Handwerker H Huang W Iyengar S Jensen MP Kennedy JD Lee N Levine J Lidster K Machin I McDermott MP McMahon SB Price TJ Ross SE Scherrer G Seal RP Sena ES Silva E Stone L Svensson CI Turk DC Whiteside G. Ensuring transparency and minimization of methodologic bias in preclinical pain research: PPRECISE considerations. PAIN 2016;157:901–9. 26683237 10.1097/j.pain.0000000000000458 PMC4794131

[3] Antunes-Martins A Perkins JR Lees J Hildebrandt T Orengo C Bennett DL. Systems biology approaches to finding novel pain mediators. Wiley Interdiscip Rev Syst Biol Med 2013;5:11–35. 23059966 10.1002/wsbm.1192

[4] Assarsson E Lundberg M Holmquist G Bjorkesten J Thorsen SB Ekman D Eriksson A Rennel Dickens E Ohlsson S Edfeldt G Andersson AC Lindstedt P Stenvang J Gullberg M Fredriksson S. Homogenous 96-plex PEA immunoassay exhibiting high sensitivity, specificity, and excellent scalability. PLoS One 2014;9:e95192. 24755770 10.1371/journal.pone.0095192 PMC3995906

[5] Baron R Maier C Attal N Binder A Bouhassira D Cruccu G Finnerup NB Haanpaa M Hansson P Hullemann P Jensen TS Freynhagen R Kennedy JD Magerl W Mainka T Reimer M Rice AS Segerdahl M Serra J Sindrup S Sommer C Tolle T Vollert J Treede RD. Peripheral neuropathic pain: a mechanism-related organizing principle based on sensory profiles. PAIN 2017;158:261–72. 27893485 10.1097/j.pain.0000000000000753 PMC5266425

[6] Bastien CH Vallières A Morin CM. Validation of the Insomnia Severity Index as an outcome measure for insomnia research. Sleep Med 2001;2:297–307. 11438246 10.1016/s1389-9457(00)00065-4

[7] Begley CG Buchan AM Dirnagl U. Robust research: institutions must do their part for reproducibility. Nature 2015;525:25–7. 26333454 10.1038/525025a

[8] Bespalov A Barnett AG Begley CG. Industry is more alarmed about reproducibility than academia. Nature 2018;563:626. 10.1038/d41586-018-07549-w 30487623

[9] Bouhassira D Attal N Alchaar H Boureau F Brochet B Bruxelle J Cunin G Fermanian J Ginies P Grun-Overdyking A Jafari-Schluep H Lantéri-Minet M Laurent B Mick G Serrie A Valade D Vicaut E. Comparison of pain syndromes associated with nervous or somatic lesions and development of a new neuropathic pain diagnostic questionnaire (DN4). PAIN 2005;114:29–36. 15733628 10.1016/j.pain.2004.12.010

[10] Bouhassira D Attal N Fermanian J Alchaar H Gautron M Masquelier E Rostaing S Lanteri-Minet M Collin E Grisart J Boureau F. Development and validation of the neuropathic pain symptom inventory. PAIN 2004;108:248–57. 15030944 10.1016/j.pain.2003.12.024

[11] Bouhassira D Letanoux M Hartemann A. Chronic pain with neuropathic characteristics in diabetic patients: a French cross-sectional study. PLoS One 2013;8:e74195. 24058527 10.1371/journal.pone.0074195 PMC3772849

[12] Bril V Perkins BA. Validation of the Toronto clinical scoring system for diabetic polyneuropathy. Diabetes Care 2002;25:2048–52. 12401755 10.2337/diacare.25.11.2048

[13] Bäckryd E Ghafouri B Carlsson AK Olausson P Gerdle B. Multivariate proteomic analysis of the cerebrospinal fluid of patients with peripheral neuropathic pain and healthy controls - a hypothesis-generating pilot study. J Pain Res 2015;8:321–33. 26170714 10.2147/JPR.S82970 PMC4492642

[14] Bäckryd E Lind AL Thulin M Larsson A Gerdle B Gordh T. High levels of cerebrospinal fluid chemokines point to the presence of neuroinflammation in peripheral neuropathic pain: a cross-sectional study of 2 cohorts of patients compared with healthy controls. PAIN 2017;158:2487–95. 28930774 10.1097/j.pain.0000000000001061 PMC5690569

[15] Bäckryd E Persson EB Larsson AI Fischer MR Gerdle B. Chronic pain patients can be classified into four groups: clustering-based discriminant analysis of psychometric data from 4665 patients referred to a multidisciplinary pain centre (a SQRP study). PLoS One 2018;13:e0192623. 29420607 10.1371/journal.pone.0192623 PMC5805304

[16] Bäckryd E Tanum L Lind AL Larsson A Gordh T. Evidence of both systemic inflammation and neuroinflammation in fibromyalgia patients, as assessed by a multiplex protein panel applied to the cerebrospinal fluid and to plasma. J Pain Res 2017;10:515–25. 28424559 10.2147/JPR.S128508 PMC5344444

[17] Comte B Baumbach J Benis A Basílio J Debeljak N Å Flobak Franken C Harel N He F Kuiper M Méndez Pérez JA Pujos-Guillot E Režen T Rozman D Schmid JA Scerri J Tieri P Van Steen K Vasudevan S Watterson S Schmidt H. Network and systems medicine: position paper of the European collaboration on science and technology action on open multiscale systems medicine. Netw Syst Med 2020;3:67–90. 32954378 10.1089/nsm.2020.0004 PMC7500076

[18] Cooke AA Connaughton RM Lyons CL McMorrow AM Roche HM. Fatty acids and chronic low grade inflammation associated with obesity and the metabolic syndrome. Eur J Pharmacol 2016;785:207–14. 27083551 10.1016/j.ejphar.2016.04.021

[19] de Williams AC Davies HT Chadury Y. Simple pain rating scales hide complex idiosyncratic meanings. PAIN 2000;85:457–63. 10781919 10.1016/S0304-3959(99)00299-7

[20] Doupis J Lyons TE Wu S Gnardellis C Dinh T Veves A. Microvascular reactivity and inflammatory cytokines in painful and painless peripheral diabetic neuropathy. J Clin Endocrinol Metab 2009;94:2157–63. 19276232 10.1210/jc.2008-2385 PMC2690431

[21] Ericson H Abu Hamdeh S Freyhult E Stiger F Backryd E Svenningsson A Gordh T Kultima K. Cerebrospinal fluid biomarkers of inflammation in trigeminal neuralgia patients operated with microvascular decompression. PAIN 2019;160:2603–11. 31373951 10.1097/j.pain.0000000000001649

[22] Eriksson L Byrne T Johansson E Trygg J Vikström C. Multi- and Megavariate Data Analysis: Basic Principles and Applications: Malmö: MKS Umetrics AB, 2013.

[23] Feldman EL Callaghan BC Pop-Busui R Zochodne DW Wright DE Bennett DL Bril V Russell JW Viswanathan V. Diabetic neuropathy. Nat Rev Dis Primers 2019;5:42. 31197183 10.1038/s41572-019-0097-9 PMC7096070

[24] Finnerup NB Attal N Haroutounian S McNicol E Baron R Dworkin RH Gilron I Haanpaa M Hansson P Jensen TS Kamerman PR Lund K Moore A Raja SN Rice AS Rowbotham M Sena E Siddall P Smith BH Wallace M. Pharmacotherapy for neuropathic pain in adults: a systematic review and meta-analysis. Lancet Neurol 2015;14:162–73. 25575710 10.1016/S1474-4422(14)70251-0 PMC4493167

[25] Finnerup NB Haroutounian S Kamerman P Baron R Bennett DL Bouhassira D Cruccu G Freeman R Hansson P Nurmikko T Raja SN Rice AS Serra J Smith BH Treede RD Jensen TS. Neuropathic pain: an updated grading system for research and clinical practice. PAIN 2016;157:1599–606. 27115670 10.1097/j.pain.0000000000000492 PMC4949003

[26] Freedman LP Cockburn IM Simcoe TS. The economics of reproducibility in preclinical research. Plos Biol 2015;13:e1002165. 26057340 10.1371/journal.pbio.1002165 PMC4461318

[27] Freynhagen R Baron R Gockel U Tölle TR. painDETECT: a new screening questionnaire to identify neuropathic components in patients with back pain. Curr Med Res Opin 2006;22:1911–20. 17022849 10.1185/030079906X132488

[28] Gerdle B Backryd E Falkenberg T Lundstrom E Ghafouri B. Changes in inflammatory plasma proteins from patients with chronic pain associated with treatment in an interdisciplinary multimodal rehabilitation program - an explorative multivariate pilot study. Scand J Pain 2019;20:125–38. 31584875 10.1515/sjpain-2019-0088

[29] Gerdle B Ghafouri B Ghafouri N Backryd E Gordh T. Signs of ongoing inflammation in female patients with chronic widespread pain: a multivariate, explorative, cross-sectional study of blood samples. Medicine (Baltimore) 2017;96:e6130. 28248866 10.1097/MD.0000000000006130 PMC5340439

[30] Gonçalves Dos Santos G Delay L Yaksh TL Corr M. Neuraxial cytokines in pain states. Front Immunol 2019;10:3061. 32047493 10.3389/fimmu.2019.03061 PMC6997465

[31] Grace PM Hutchinson MR Maier SF Watkins LR. Pathological pain and the neuroimmune interface. Nat Rev Immunol 2014;14:217–31. 24577438 10.1038/nri3621 PMC5525062

[32] Groh J Basu R Stanley ER Martini R. Cell-surface and secreted isoforms of CSF-1 exert opposing roles in macrophage-mediated neural damage in Cx32-deficient mice. J Neurosci 2016;36:1890–901. 26865613 10.1523/JNEUROSCI.3427-15.2016 PMC4748074

[33] Hotamisligil GS. Inflammation and metabolic disorders. Nature 2006;444:860–7. 17167474 10.1038/nature05485

[34] Hunter P. The reproducibility “crisis”: reaction to replication crisis should not stifle innovation. EMBO Rep 2017;18:1493–6. 28794201 10.15252/embr.201744876 PMC5579390

[35] Ioannidis JP. Why most published research findings are false. PLoS Med 2005;2:e124. 16060722 10.1371/journal.pmed.0020124 PMC1182327

[36] Kadetoff D Lampa J Westman M Andersson M Kosek E. Evidence of central inflammation in fibromyalgia-increased cerebrospinal fluid interleukin-8 levels. J Neuroimmunol 2012;242:33–8. 22126705 10.1016/j.jneuroim.2011.10.013

[37] Kan HW Hsieh JH Chien HF Lin YH Yeh TY Chao CC Hsieh ST. CD40-mediated HIF-1α expression underlying microangiopathy in diabetic nerve pathology. Dis Model Mech 2018;11:dmm033647. 29549140 10.1242/dmm.033647 PMC5963861

[38] Kessler JA Smith AG Cha BS Choi SH Wymer J Shaibani A Ajroud-Driss S Vinik A. Double-blind, placebo-controlled study of HGF gene therapy in diabetic neuropathy. Ann Clin Transl Neurol 2015;2:465–78. 26000320 10.1002/acn3.186 PMC4435702

[39] Kingsmore SF. Multiplexed protein measurement: technologies and applications of protein and antibody arrays. Nat Rev Drug Discov 2006;5:310–20. 16582876 10.1038/nrd2006 PMC1780251

[40] Ko KR Lee J Lee D Nho B Kim S. Hepatocyte growth factor (HGF) promotes peripheral nerve regeneration by activating repair Schwann cells. Sci Rep 2018;8:8316. 29844434 10.1038/s41598-018-26704-x PMC5973939

[41] Langjahr M Schubert AL Sommer C Üçeyler N. Increased pro-inflammatory cytokine gene expression in peripheral blood mononuclear cells of patients with polyneuropathies. J Neurol 2018;265:618–27. 29372388 10.1007/s00415-018-8748-4

[42] Lassen J Stürner KH Gierthmühlen J Dargvainiene J Kixmüller D Leypoldt F Baron R Hüllemann P. Protective role of natural killer cells in neuropathic pain conditions. PAIN 2021;162:2366–75. 33769361 10.1097/j.pain.0000000000002274

[43] Ludwig J Binder A Steinmann J Wasner G Baron R. Cytokine expression in serum and cerebrospinal fluid in non-inflammatory polyneuropathies. J Neurol Neurosurg Psychiatry 2008;79:1268–73. 18550631 10.1136/jnnp.2007.134528

[44] Lundberg M Eriksson A Tran B Assarsson E Fredriksson S. Homogeneous antibody-based proximity extension assays provide sensitive and specific detection of low-abundant proteins in human blood. Nucleic Acids Res 2011;39:e102. 21646338 10.1093/nar/gkr424 PMC3159481

[45] Macleod MR Michie S Roberts I Dirnagl U Chalmers I Ioannidis JP Al-Shahi Salman R Chan AW Glasziou P. Biomedical research: increasing value, reducing waste. Lancet 2014;383:101–4. 24411643 10.1016/S0140-6736(13)62329-6

[46] Mao J. Translational pain research: achievements and challenges. J Pain 2009;10:1001–11. 19628433 10.1016/j.jpain.2009.06.002 PMC2757533

[47] McCracken LM Dhingra L. A short version of the Pain Anxiety Symptoms Scale (PASS-20): preliminary development and validity. Pain Res Manag 2002;7:45–50. 16231066 10.1155/2002/517163

[48] Moen A Lind AL Thulin M Kamali-Moghaddam M Roe C Gjerstad J Gordh T. Inflammatory serum protein profiling of patients with lumbar radicular pain one year after disc herniation. Int J Inflam 2016;2016:3874964. 27293953 10.1155/2016/3874964 PMC4879232

[49] Monteiro R Azevedo I. Chronic inflammation in obesity and the metabolic syndrome. Mediators Inflamm 2010:2010. 10.1155/2010/289645 PMC2913796 20706689

[50] Naruse K. Schwann cells as crucial players in diabetic neuropathy. Adv Exp Med Biol 2019;1190:345–56. 31760655 10.1007/978-981-32-9636-7_22

[51] Nordberg N Ohlsson S. PEA: An enabling technology for high-multiplex protein biomarker discovery. Advancing precision medicine: Current and future proteogenomic strategies for biomarker discovery and development. Washington, DC: Science/AAAS, 2017:31.

[52] Olausson P Gerdle B Ghafouri N Sjostrom D Blixt E Ghafouri B. Protein alterations in women with chronic widespread pain—An explorative proteomic study of the trapezius muscle. Sci Rep 2015;5:11894. 26150212 10.1038/srep11894 PMC4493691

[53] Osman A Barrios FX Kopper BA Hauptmann W Jones J O'Neill E. Factor structure, reliability, and validity of the pain catastrophizing scale. J Behav Med 1997;20:589–605. 9429990 10.1023/a:1025570508954

[54] Perrin S. Preclinical research: make mouse studies work. Nature 2014;507:423–5. 24678540 10.1038/507423a

[55] Pincus T Williams AC Vogel S Field A. The development and testing of the depression, anxiety, and positive outlook scale (DAPOS). PAIN 2004;109:181–8. 15082140 10.1016/j.pain.2004.02.004

[56] Pop-Busui R Ang L Holmes C Gallagher K Feldman EL. Inflammation as a therapeutic target for diabetic neuropathies. Curr Diab Rep 2016;16:29. 26897744 10.1007/s11892-016-0727-5 PMC5127166

[57] Rosenberger DC Blechschmidt V Timmerman H Wolff A Treede RD. Challenges of neuropathic pain: focus on diabetic neuropathy. J Neural Transm (Vienna) 2020;127:589–624. 32036431 10.1007/s00702-020-02145-7 PMC7148276

[58] Salminen A. Increased immunosuppression impairs tissue homeostasis with aging and age-related diseases. J Mol Med (Berl) 2021;99:1–20. 33025106 10.1007/s00109-020-01988-7 PMC7782450

[59] Scarpellini E Tack J. Obesity and metabolic syndrome: an inflammatory condition. Dig Dis 2012;30:148–53. 22722429 10.1159/000336664

[60] Shachar I Karin N. The dual roles of inflammatory cytokines and chemokines in the regulation of autoimmune diseases and their clinical implications. J Leukoc Biol 2013;93:51–61. 22949334 10.1189/jlb.0612293

[61] Shillo P Sloan G Greig M Hunt L Selvarajah D Elliott J Gandhi R Wilkinson ID Tesfaye S. Painful and painless diabetic neuropathies: what is the difference?. Curr Diab Rep 2019;19:32. 31065863 10.1007/s11892-019-1150-5 PMC6505492

[62] Sloan G Shillo P Selvarajah D Wu J Wilkinson ID Tracey I Anand P Tesfaye S. A new look at painful diabetic neuropathy. Diabetes Res Clin Pract 2018;144:177–91. 30201394 10.1016/j.diabres.2018.08.020

[63] Sommer C Leinders M Üçeyler N. Inflammation in the pathophysiology of neuropathic pain. PAIN 2018;159:595–602. 29447138 10.1097/j.pain.0000000000001122

[64] Strom A Brüggemann J Ziegler I Jeruschke K Weiss J Al-Hasani H Roden M Ziegler D. Pronounced reduction of cutaneous Langerhans cell density in recently diagnosed type 2 diabetes. Diabetes 2014;63:1148–53. 24319115 10.2337/db13-1444

[65] Tan G Jensen MP Thornby JI Shanti BF. Validation of the Brief pain inventory for chronic nonmalignant pain. J Pain 2004;5:133–7. 15042521 10.1016/j.jpain.2003.12.005

[66] Themistocleous AC Ramirez JD Shillo PR Lees JG Selvarajah D Orengo C Tesfaye S Rice AS Bennett DL. The Pain in Neuropathy Study (PiNS): a cross-sectional observational study determining the somatosensory phenotype of painful and painless diabetic neuropathy. PAIN 2016;157:1132–45. 27088890 10.1097/j.pain.0000000000000491 PMC4834814

[67] Tracy LM. Psychosocial factors and their influence on the experience of pain. Pain Rep 2017;2:e602. 29392217 10.1097/PR9.0000000000000602 PMC5741357

[68] Van Acker K Bouhassira D De Bacquer D Weiss S Matthys K Raemen H Mathieu C Colin IM. Prevalence and impact on quality of life of peripheral neuropathy with or without neuropathic pain in type 1 and type 2 diabetic patients attending hospital outpatients clinics. Diabetes Metab 2009;35:206–13. 19297223 10.1016/j.diabet.2008.11.004

[69] Wheelock AM Wheelock CE. Trials and tribulations of 'omics data analysis: assessing quality of SIMCA-based multivariate models using examples from pulmonary medicine. Mol Biosyst 2013;9:2589–96. 23999822 10.1039/c3mb70194h

[70] Yu X Basbaum A Guan Z. Contribution of colony-stimulating factor 1 to neuropathic pain. Pain Rep 2021;6:e883. 33981926 10.1097/PR9.0000000000000883 PMC8108585

[71] Ziegler D. [Diabetic polyneuropathy]. Internist (Berl) 2020;61:243–53. 32086529 10.1007/s00108-020-00770-8

[72] Ziegler D Strom A Bönhof GJ Kannenberg JM Heier M Rathmann W Peters A Meisinger C Roden M Thorand B Herder C. Deficits in systemic biomarkers of neuroinflammation and growth factors promoting nerve regeneration in patients with type 2 diabetes and polyneuropathy. BMJ Open Diabetes Res Care 2019;7:e000752. 10.1136/bmjdrc-2019-000752 PMC6887496 31803481

