# Supplementary material for: Hepatocyte growth factor, colony-stimulating factor 1, CD40, and 11 other inflammation-related proteins are associated with pain in diabetic neuropathy: exploration and replication serum data from the Pain in Neuropathy Study
Source: Pain. 2021 Aug 24;163(5):897–909. doi: 10.1097/j.pain.0000000000002451 (PMC9009322; doi:10.1097/j.pain.0000000000002451)
Supplement: Supplementary file 2 [file jop-163-897-s002.docx]

# Supplemental Digital Content 1

Complete list of the studied 92 inflammation-related proteins, with abbreviation and followed by UniProt ID.

1. Adenosine Deaminase (ADA) P00813
2. Artemin (ARTN) Q5T4W7
3. Axin-1 (AXIN1) O15169
4. Beta-nerve growth factor (Beta-NGF) P01138
5. Caspase-8 (CASP-8) Q14790
6. C-C motif chemokine 3 (CCL3) P10147
7. C-C motif chemokine 4 (CCL4) P13236
8. C-C motif chemokine 19 (CCL19) Q99731
9. C-C motif chemokine 20 (CCL20) P78556
10. C-C motif chemokine 23 (CCL23) P55773
11. C-C motif chemokine 25 (CCL25) O15444
12. C-C motif chemokine 28 (CCL28) Q9NRJ3
13. CD40L receptor (CD40) P25942
14. CUB domain-containing protein 1 (CDCP1) Q9H5V8
15. C-X-C motif chemokine 1 (CXCL1) P09341
16. C-X-C motif chemokine 5 (CXCL5) P42830
17. C-X-C motif chemokine 6 (CXCL6) P80162
18. C-X-C motif chemokine 9 (CXCL9) Q07325
19. C-X-C motif chemokine 10 (CXCL10) P02778
20. C-X-C motif chemokine 11 (CXCL11) O14625
21. Cystatin D (CST5) P28325
22. Delta and Notch-like epidermal growth factor-related receptor (DNER) Q8NFT8
23. Eotaxin (CCL11) P51671
24. Eukaryotic translation initiation factor 4E-binding protein 1 (4E-BP1) Q13541
25. Fibroblast growth factor 21 (FGF-21) Q9NSA1
26. Fibroblast growth factor 23 (FGF-23) Q9GZV9
27. Fibroblast growth factor 5 (FGF-5) P12034
28. Fibroblast growth factor 19 (FGF-19) O95750
29. Fms-related tyrosine kinase 3 ligand (Flt3L) P49771
30. Fractalkine (CX3CL1) P78423
31. Glial cell line-derived neurotrophic factor (GDNF) P39905
32. Hepatocyte growth factor (HGF) P14210
33. Interferon gamma (IFN-gamma) P01579
34. Interleukin-1 alpha (IL-1 alpha) P01583
35. Interleukin-2 (IL-2) P60568
36. Interleukin-2 receptor subunit beta (IL-2RB) P14784
37. Interleukin-4 (IL-4) P05112
38. Interleukin-5 (IL5) P05113
39. Interleukin-6 (IL6) P05231
40. Interleukin-7 (IL-7) P13232
41. Interleukin-8 (IL-8) P10145
42. Interleukin-10 (IL10) P22301
43. Interleukin-10 receptor subunit alpha (IL-10RA) Q13651
44. Interleukin-10 receptor subunit beta (IL-10RB) Q08334
45. Interleukin-12 subunit beta (IL-12B) P29460
46. Interleukin-13 (IL-13) P35225
47. Interleukin-15 receptor subunit alpha (IL-15RA) Q13261
48. Interleukin-17A (IL-17A) Q16552
49. Interleukin-17C (IL-17C) Q9P0M4
50. Interleukin-18 (IL-18) Q14116
51. Interleukin-18 receptor 1 (IL-18R1) Q13478
52. Interleukin-20 (IL-20) Q9NYY1
53. Interleukin-20 receptor subunit alpha (IL-20RA) Q9UHF4
54. Interleukin-22 receptor subunit alpha-1 (IL-22 RA1) Q8N6P7
55. Interleukin-24 (IL-24) Q13007
56. Interleukin-33 (IL-33) O95760
57. Latency-associated peptide transforming growth factor beta-1 (LAP TGF-beta-1) P01137
58. Leukemia inhibitory factor (LIF) P15018
59. Leukemia inhibitory factor receptor (LIF-R) P42702
60. Macrophage colony-stimulating factor 1 (CSF-1) P09603
61. Matrix metalloproteinase-1 (MMP-1) P03956
62. Matrix metalloproteinase-10 (MMP-10) P09238
63. Monocyte chemotactic protein 1 (MCP-1) P13500
64. Monocyte chemotactic protein 2 (MCP-2) P80075
65. Monocyte chemotactic protein 3 (MCP-3) P80098
66. Monocyte chemotactic protein 4 (MCP-4) Q99616
67. Natural killer cell receptor 2B4 (CD244) Q9BZW8
68. Neurotrophin-3 (NT-3) P20783
69. Neurturin (NRTN) Q99748
70. Oncostatin-M (OSM) P13725
71. Osteoprotegerin (OPG) O00300
72. Programmed cell death 1 ligand 1 (PD-L1) Q9NZQ7
73. Protein S100-A12 (EN-RAGE) P80511
74. Signaling lymphocytic activation molecule (SLAMF1) Q13291
75. SIR2-like protein 2 (SIRT2) Q8IXJ6
76. STAM-binding protein (STAMBP) O95630
77. Stem cell factor (SCF) P21583
78. Sulfotransferase 1A1 (ST1A1) P50225
79. T cell surface glycoprotein CD6 isoform (CD6) P30203
80. T-cell surface glycoprotein CD5 (CD5) P06127
81. T-cell surface glycoprotein CD8 alpha chain (CD8A) P01732
82. Thymic stromal lymphopoietin (TSLP) Q969D9
83. TNF-beta (TNFB) P01374
84. TNF-related activation-induced cytokine (TRANCE) O14788
85. TNF-related apoptosis-inducing ligand (TRAIL) P50591
86. Transforming growth factor alpha (TGF-alpha) P01135
87. Tumor necrosis factor (Ligand) superfamily, member 12 (TWEAK) O43508
88. Tumor necrosis factor (TNF) P01375
89. Tumor necrosis factor ligand superfamily member 14 (TNFSF14) O43557
90. Tumor necrosis factor receptor superfamily member 9 (TNFRSF9) Q07011
91. Urokinase-type plasminogen activator (uPA) P00749
92. Vascular endothelial growth factor A (VEGF-A) P15692

# Supplemental Digital Content 2

Dendrograms representing three groups of patients in the hierarchical cluster analysis (HCA). The vertical scale (Y-axis) is a similarity/dissimilarity measure, with similarity increasing as the numerical value of the scale approaches zero [1]. The individual observations (patients) are on the bottom row (X-axis). Note the similarities between the dendrograms of the exploratory and replication cohorts.

## Dendrogram Exploratory cohort


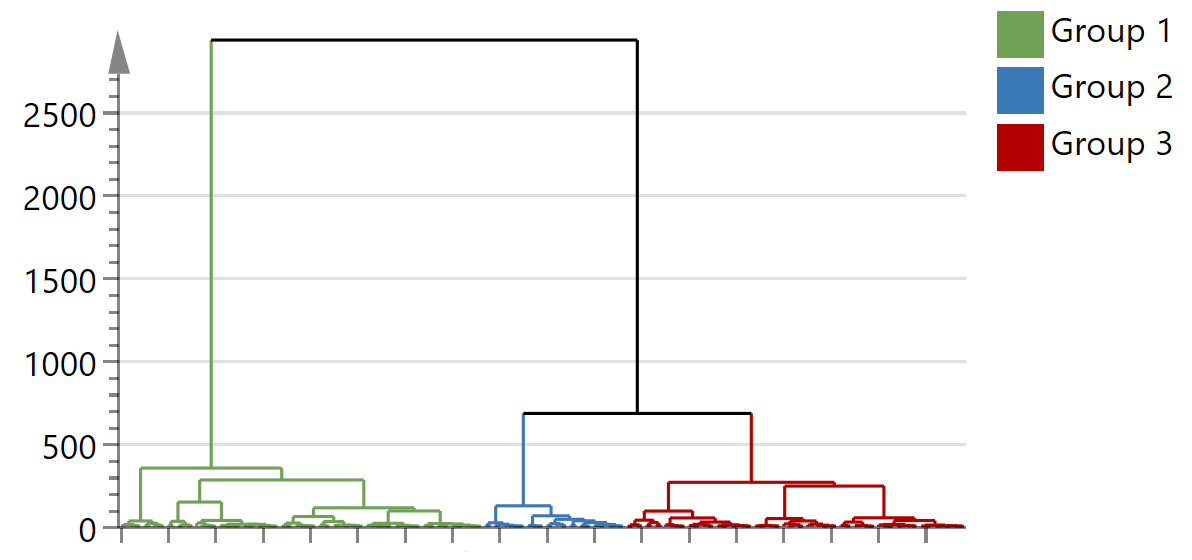


## Dendrogram Replication cohort


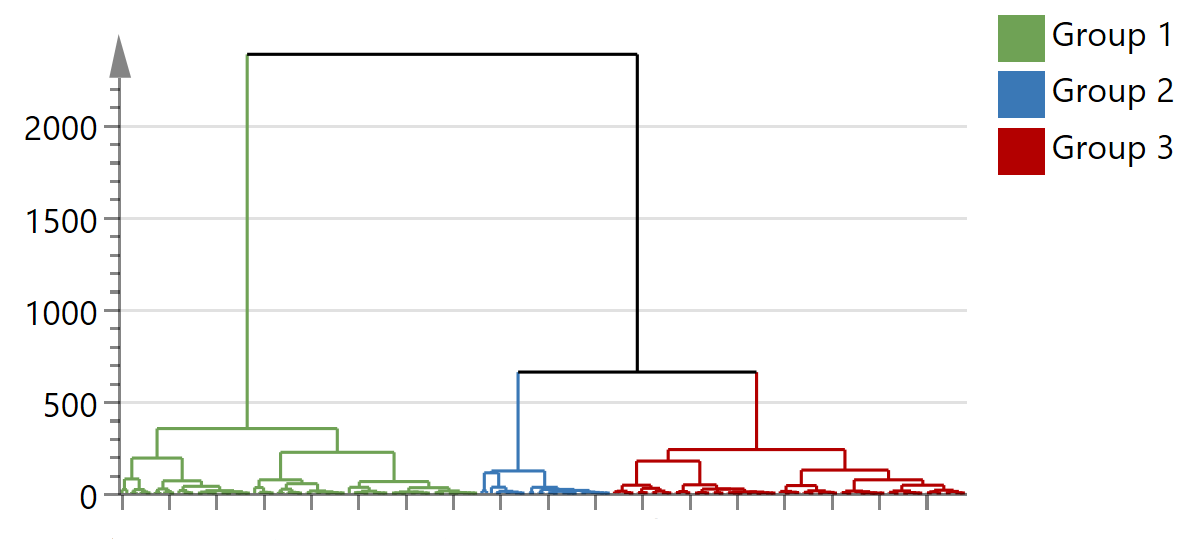


## References

[1] Eriksson L, Byrne T, Johansson E, Trygg J, Vikström C. Multi- and Megavariate Data Analysis: Basic Principles and Applications. Malmö: MKS Umetrics AB, 2013.

# Supplemental Digital Content 3

**Exploratory cohort:** Medians and interquartile range for Groups 1, 2, and 3 for all 74 proteins. Data are expressed as normalized protein expression (NPX). Values of NPX are on a log2 scale. Furthest to the right we report P-values from the omnibus test (Kruskal Wallis test).

|  | **Group 1** | | |  | **Group 2** | | |  | **Group 3** | | |  |
| --- | --- | --- | --- | --- | --- | --- | --- | --- | --- | --- | --- | --- |
|  | 25th | 50th | 75th |  | 25th | 50th | 75th |  | 25th | 50th | 75th | **P-value** |
| 4E-BP1 | 6.62 | 7.35 | 8.07 |  | 7.87 | 8.28 | 8.90 |  | 7.00 | 7.69 | 8.31 | 6.1E-05 |
| ADA | 3.18 | 3.39 | 3.67 |  | 3.72 | 3.92 | 4.19 |  | 3.58 | 3.71 | 3.98 | 1.21E-10 |
| AXIN1 | 1.04 | 1.30 | 1.72 |  | 1.79 | 2.05 | 2.47 |  | 1.42 | 1.70 | 2.16 | 6.85E-11 |
| Beta-NGF | 1.56 | 1.72 | 1.91 |  | 2.18 | 2.29 | 2.47 |  | 1.86 | 2.05 | 2.21 | 3.04E-16 |
| CASP-8 | 1.10 | 1.26 | 1.49 |  | 1.80 | 2.10 | 2.32 |  | 1.50 | 1.64 | 1.96 | 9.74E-20 |
| CCL11 | 7.99 | 8.15 | 8.42 |  | 8.68 | 8.84 | 9.14 |  | 8.29 | 8.62 | 8.80 | 4.33E-14 |
| CCL19 | 8.75 | 9.11 | 9.60 |  | 9.56 | 10.10 | 10.44 |  | 9.13 | 9.55 | 10.07 | 1.18E-07 |
| CCL20 | 4.42 | 5.00 | 5.59 |  | 5.53 | 5.90 | 7.41 |  | 4.93 | 5.42 | 6.38 | 1.25E-07 |
| CCL23 | 9.48 | 9.80 | 10.04 |  | 10.35 | 10.66 | 10.98 |  | 9.87 | 10.06 | 10.52 | 9.51E-14 |
| CCL25 | 6.15 | 6.66 | 6.85 |  | 6.93 | 7.11 | 7.34 |  | 6.45 | 6.75 | 7.01 | 4.28E-06 |
| CCL28 | 1.19 | 1.38 | 1.51 |  | 1.80 | 1.95 | 2.16 |  | 1.46 | 1.67 | 1.85 | 2.09E-16 |
| CCL3 | 4.72 | 5.05 | 5.36 |  | 5.77 | 6.17 | 6.31 |  | 5.44 | 5.71 | 6.03 | 2.71E-16 |
| CCL4 | 6.52 | 6.91 | 7.25 |  | 7.46 | 8.00 | 8.28 |  | 7.19 | 7.59 | 7.89 | 1.65E-13 |
| CD244 | 5.25 | 5.48 | 5.72 |  | 5.99 | 6.22 | 6.38 |  | 5.74 | 5.90 | 6.14 | 7.29E-19 |
| CD40 | 9.36 | 9.59 | 9.82 |  | 10.26 | 10.51 | 10.77 |  | 9.80 | 10.03 | 10.25 | 3.1E-19 |
| CD5 | 4.50 | 4.72 | 4.95 |  | 5.39 | 5.73 | 5.84 |  | 4.87 | 5.16 | 5.35 | 4.84E-19 |
| CD6 | 3.84 | 4.16 | 4.39 |  | 4.77 | 4.92 | 5.30 |  | 4.43 | 4.64 | 5.02 | 4.31E-19 |
| CDCP1 | 2.95 | 3.27 | 3.64 |  | 3.30 | 3.68 | 4.24 |  | 3.37 | 3.79 | 4.18 | 8.6E-07 |
| CSF-1 | 7.58 | 7.75 | 7.88 |  | 8.12 | 8.26 | 8.34 |  | 7.88 | 8.01 | 8.08 | 3.76E-22 |
| CST5 | 5.61 | 5.99 | 6.30 |  | 6.09 | 6.59 | 7.00 |  | 5.85 | 6.21 | 6.66 | 5.3E-05 |
| CX3CL1 | 5.34 | 5.67 | 5.89 |  | 6.17 | 6.39 | 6.65 |  | 5.86 | 6.00 | 6.18 | 7.76E-17 |
| CXCL1 | 7.68 | 8.07 | 8.66 |  | 8.96 | 9.32 | 9.52 |  | 8.44 | 8.78 | 9.19 | 2.41E-14 |
| CXCL10 | 7.89 | 8.22 | 8.55 |  | 8.83 | 9.23 | 9.83 |  | 8.29 | 8.80 | 9.29 | 1.12E-10 |
| CXCL11 | 7.23 | 7.64 | 8.14 |  | 8.58 | 8.78 | 9.29 |  | 8.04 | 8.57 | 8.95 | 3.09E-17 |
| CXCL5 | 11.18 | 11.81 | 12.22 |  | 12.28 | 12.67 | 13.18 |  | 11.80 | 12.22 | 12.75 | 3.1E-09 |
| CXCL6 | 7.73 | 8.11 | 8.58 |  | 9.02 | 9.38 | 9.68 |  | 8.51 | 8.92 | 9.32 | 5.25E-18 |
| CXCL9 | 7.32 | 7.72 | 8.35 |  | 8.09 | 8.96 | 9.74 |  | 7.78 | 8.06 | 8.68 | 1.25E-06 |
| DNER | 7.44 | 7.71 | 7.96 |  | 7.99 | 8.19 | 8.38 |  | 7.77 | 8.00 | 8.17 | 6.69E-11 |
| EN-RAGE | 3.38 | 4.05 | 4.54 |  | 5.71 | 6.29 | 6.55 |  | 4.39 | 5.02 | 5.67 | 6.29E-18 |
| FGF-19 | 6.81 | 7.38 | 8.00 |  | 8.00 | 8.49 | 8.87 |  | 7.28 | 7.90 | 8.43 | 6.4E-07 |
| FGF-21 | 5.18 | 6.06 | 6.78 |  | 6.06 | 7.11 | 8.76 |  | 5.52 | 6.84 | 7.61 | 0.001119 |
| FGF-23 | 1.08 | 1.36 | 1.70 |  | 1.76 | 2.00 | 2.65 |  | 1.31 | 1.60 | 1.97 | 1.07E-07 |
| FGF-5 | 0.99 | 1.13 | 1.26 |  | 1.42 | 1.63 | 1.73 |  | 1.20 | 1.32 | 1.45 | 3.05E-14 |
| Flt3L | 8.40 | 8.76 | 9.12 |  | 8.98 | 9.27 | 9.50 |  | 8.86 | 9.20 | 9.49 | 9.95E-08 |
| GDNF | 1.62 | 1.77 | 1.95 |  | 2.22 | 2.35 | 2.41 |  | 1.91 | 2.06 | 2.22 | 3.97E-13 |
| HGF | 8.43 | 8.65 | 8.99 |  | 9.47 | 9.67 | 9.91 |  | 9.02 | 9.23 | 9.47 | 5.87E-20 |
| IL10 | 2.33 | 2.51 | 2.80 |  | 3.16 | 3.41 | 3.68 |  | 2.78 | 2.93 | 3.28 | 9.57E-19 |
| IL-10RA | 0.88 | 0.98 | 1.17 |  | 1.06 | 1.30 | 1.39 |  | 1.01 | 1.16 | 1.48 | 6.27E-05 |
| IL-10RB | 6.68 | 6.90 | 7.10 |  | 7.35 | 7.51 | 7.66 |  | 6.97 | 7.19 | 7.35 | 2.41E-16 |
| IL-12B | 4.01 | 4.42 | 4.65 |  | 4.73 | 5.18 | 5.53 |  | 4.28 | 4.67 | 4.98 | 4.76E-07 |
| IL-15RA | 0.70 | 0.79 | 0.90 |  | 1.10 | 1.19 | 1.28 |  | 0.89 | 1.03 | 1.09 | 1.67E-18 |
| IL-17A | 0.79 | 1.00 | 1.27 |  | 1.31 | 1.56 | 2.12 |  | 0.98 | 1.11 | 1.42 | 8.62E-08 |
| IL-17C | 1.41 | 1.81 | 2.20 |  | 2.07 | 2.41 | 3.36 |  | 1.44 | 1.86 | 2.22 | 3.06E-06 |
| IL18 | 7.38 | 7.92 | 8.28 |  | 8.40 | 8.73 | 9.13 |  | 7.99 | 8.22 | 8.61 | 3.68E-13 |
| IL-18R1 | 6.73 | 7.12 | 7.43 |  | 7.40 | 7.71 | 8.03 |  | 7.23 | 7.46 | 7.82 | 1.89E-09 |
| IL6 | 3.25 | 3.59 | 4.13 |  | 4.07 | 4.54 | 5.33 |  | 3.54 | 4.07 | 4.65 | 1.54E-07 |
| IL7 | 4.58 | 4.94 | 5.29 |  | 5.23 | 5.53 | 5.82 |  | 4.99 | 5.46 | 5.82 | 2.26E-08 |
| IL8 | 6.58 | 6.95 | 7.22 |  | 7.28 | 7.85 | 8.29 |  | 7.21 | 7.52 | 7.92 | 9.98E-13 |
| LAP TGF-beta-1 | 7.45 | 7.73 | 7.92 |  | 8.30 | 8.47 | 8.79 |  | 7.97 | 8.21 | 8.39 | 4.88E-20 |
| LIF-R | 2.78 | 2.97 | 3.11 |  | 3.41 | 3.57 | 3.69 |  | 3.13 | 3.26 | 3.44 | 1.03E-18 |
| MCP-1 | 10.29 | 10.73 | 11.01 |  | 11.52 | 11.71 | 11.97 |  | 10.87 | 11.17 | 11.50 | 6.48E-18 |
| MCP-2 | 7.97 | 8.46 | 8.79 |  | 9.18 | 9.54 | 9.73 |  | 8.70 | 9.05 | 9.45 | 9E-15 |
| MCP-3 | 1.86 | 2.09 | 2.36 |  | 2.70 | 2.93 | 3.21 |  | 2.20 | 2.41 | 2.75 | 9.43E-14 |
| MCP-4 | 4.03 | 4.40 | 4.80 |  | 5.44 | 5.71 | 6.00 |  | 4.55 | 5.09 | 5.57 | 4.69E-15 |
| MMP-1 | 13.27 | 13.79 | 14.19 |  | 13.99 | 14.23 | 14.95 |  | 13.46 | 14.17 | 14.49 | 4.73E-05 |
| MMP-10 | 5.65 | 5.97 | 6.31 |  | 6.47 | 7.05 | 7.34 |  | 5.93 | 6.47 | 6.91 | 3.1E-10 |
| NT-3 | 1.20 | 1.36 | 1.53 |  | 1.59 | 1.78 | 2.15 |  | 1.46 | 1.61 | 1.90 | 2.24E-12 |
| OPG | 10.30 | 10.52 | 10.85 |  | 10.79 | 11.06 | 11.28 |  | 10.66 | 10.96 | 11.21 | 7.81E-10 |
| OSM | 2.94 | 3.58 | 4.24 |  | 4.49 | 5.01 | 5.43 |  | 3.93 | 4.27 | 4.62 | 4.06E-12 |
| PD-L1 | 3.66 | 3.84 | 4.03 |  | 4.51 | 4.71 | 4.99 |  | 4.10 | 4.26 | 4.48 | 1.86E-20 |
| SCF | 9.49 | 9.79 | 10.00 |  | 9.90 | 10.23 | 10.39 |  | 9.67 | 9.98 | 10.20 | 1.11E-05 |
| SIRT2 | 1.41 | 1.62 | 1.96 |  | 2.19 | 2.43 | 2.77 |  | 1.77 | 2.16 | 2.52 | 4.22E-11 |
| SLAMF1 | 1.75 | 2.03 | 2.33 |  | 2.31 | 2.85 | 3.18 |  | 2.07 | 2.35 | 2.70 | 4.94E-11 |
| ST1A1 | 0.79 | 0.98 | 1.32 |  | 1.56 | 1.82 | 2.29 |  | 1.11 | 1.54 | 2.23 | 2.47E-11 |
| STAMPB | 2.77 | 3.08 | 3.47 |  | 3.72 | 3.94 | 4.29 |  | 3.28 | 3.62 | 4.13 | 9.1E-12 |
| TGF-alpha | 2.97 | 3.26 | 3.67 |  | 4.29 | 4.76 | 5.22 |  | 3.56 | 3.94 | 4.34 | 1.24E-18 |
| TNFB | 3.37 | 3.63 | 3.84 |  | 4.01 | 4.13 | 4.37 |  | 3.61 | 3.92 | 4.18 | 2.35E-10 |
| TNFRSF9 | 5.80 | 6.05 | 6.41 |  | 6.80 | 7.14 | 7.31 |  | 6.25 | 6.46 | 6.79 | 2.52E-13 |
| TNFSF14 | 3.93 | 4.26 | 4.69 |  | 5.33 | 5.61 | 5.98 |  | 4.53 | 4.88 | 5.47 | 7.35E-16 |
| TRAIL | 7.73 | 7.99 | 8.17 |  | 8.52 | 8.71 | 8.87 |  | 8.06 | 8.30 | 8.46 | 3.64E-16 |
| TRANCE | 3.48 | 3.89 | 4.21 |  | 4.07 | 4.36 | 5.06 |  | 3.90 | 4.20 | 4.64 | 4.98E-07 |
| TWEAK | 8.89 | 9.25 | 9.55 |  | 9.80 | 10.05 | 10.16 |  | 9.45 | 9.67 | 9.88 | 7.9E-15 |
| uPA | 9.70 | 9.92 | 10.11 |  | 10.25 | 10.52 | 10.75 |  | 10.09 | 10.31 | 10.49 | 5.49E-14 |
| VEGFA | 9.63 | 9.97 | 10.31 |  | 10.49 | 10.85 | 11.08 |  | 10.13 | 10.36 | 10.79 | 1.83E-14 |

# Supplemental Digital Content 4

Here we present OPLS-DA results for both the exploratory and the replication cohorts. In both OPLS-DA models, Group 2 is compared to Group (1+3).

OPLS-DA characteristics of:

- the exploratory cohort: n=179, 1 predictive component, R^2^=0.46, Q^2^=0.45, p<0.001 by CV-ANOVA
- the replication cohort: n=179, 1 predictive component, R^2^=0.45, Q^2^=0.43, p<0.001 by CV-ANOVA

In the table below, proteins are listed in falling order of p(corr), i.e., in falling order of importance for group discrimination (Group 2 vs Group (1+3)). To illustrate the overlap in results between the two OPLS-DA models, the top 10 proteins of the exploratory cohort are marked in red in both columns of the table. Likewise, the top 11-20 proteins of the exploratory cohort are marked in blue in both columns. Hence, 70 % of the top 20 proteins in the exploratory cohort are among the top 20 proteins of the replication cohort. Moreover, the top 3 proteins are the same in both cohorts.

| Exploratory cohort | | Replication cohort | |
| --- | --- | --- | --- |
| **Protein** | **p(corr)** | **Protein** | **p(corr)** |
| **CSF-1** | 0.842721 | **HGF** | 0.830767 |
| **HGF** | 0.802543 | **CSF-1** | 0.799886 |
| **CD40** | 0.795836 | **CD40** | 0.789346 |
| **LAP TGF-beta-1** | 0.77131 | **PD-L1** | 0.78381 |
| **CD5** | 0.7681 | **IL-15RA** | 0.742313 |
| **MCP-1** | 0.76344 | **CCL3** | 0.736309 |
| **TGF-alpha** | 0.756069 | **LAP TGF-beta-1** | 0.734514 |
| **CASP-8** | 0.736799 | **LIF-R** | 0.723062 |
| **CD244** | 0.736494 | **IL-10RB** | 0.713833 |
| **EN-RAGE** | 0.736031 | **MCP-1** | 0.707186 |
| **LIF-R** | 0.724593 | **TGF-alpha** | 0.694755 |
| **IL-10RB** | 0.718202 | **CXCL6** | 0.68166 |
| **PD-L1** | 0.704166 | **TNFRSF9** | 0.679991 |
| **CD6** | 0.69874 | **Beta-NGF** | 0.675636 |
| **TNFSF14** | 0.695835 | **IL-18R1** | 0.672991 |
| **CXCL6** | 0.689267 | **TNFSF14** | 0.65982 |
| **CX3CL1** | 0.669659 | **VEGFA** | 0.659149 |
| **TRAIL** | 0.661307 | **CD5** | 0.635995 |
| **OSM** | 0.659455 | **CASP-8** | 0.63188 |
| **Beta-NGF** | 0.655565 | **uPA** | 0.629128 |
| **VEGFA** | 0.652161 | **CXCL11** | 0.628442 |
| **TWEAK** | 0.646131 | **MCP-4** | 0.61857 |
| **CCL28** | 0.640505 | **CCL11** | 0.613213 |
| **IL18** | 0.640338 | **CCL4** | 0.612208 |
| **CXCL11** | 0.638898 | **TRAIL** | 0.611102 |
| **CCL23** | 0.636728 | **IL10** | 0.608897 |
| **IL10** | 0.635433 | **CXCL1** | 0.599445 |
| **MCP-2** | 0.63482 | **CCL23** | 0.598522 |
| **MCP-4** | 0.634224 | **CD244** | 0.597053 |
| **TNFRSF9** | 0.619853 | **OSM** | 0.583133 |
| **CXCL1** | 0.616724 | **CX3CL1** | 0.582003 |
| **CCL11** | 0.611733 | **MCP-3** | 0.579831 |
| **FGF-5** | 0.60451 | **IL8** | 0.579176 |
| **MCP-3** | 0.576551 | **MCP-2** | 0.573302 |
| **uPA** | 0.575872 | **TWEAK** | 0.56792 |
| **STAMPB** | 0.572495 | **IL7** | 0.566388 |
| **GDNF** | 0.564567 | **OPG** | 0.546209 |
| **CCL4** | 0.564319 | **CXCL10** | 0.539709 |
| **SLAMF1** | 0.563296 | **GDNF** | 0.537127 |
| **IL-18R1** | 0.560489 | **IL18** | 0.529563 |
| **IL-15RA** | 0.552546 | **CCL19** | 0.527195 |
| **CCL3** | 0.549905 | **IL-12B** | 0.521557 |
| **CCL19** | 0.544847 | **SLAMF1** | 0.517712 |
| **IL8** | 0.538197 | **IL6** | 0.513501 |
| **CXCL10** | 0.53688 | **CDCP1** | 0.490433 |
| **SIRT2** | 0.524277 | **EN-RAGE** | 0.484695 |
| **AXIN1** | 0.523795 | **CXCL9** | 0.479509 |
| **MMP-10** | 0.523113 | **ADA** | 0.478856 |
| **OPG** | 0.513372 | **STAMBP** | 0.464267 |
| **CXCL5** | 0.51293 | **CCL28** | 0.455634 |
| **ADA** | 0.50979 | **CD8A** | 0.438921 |
| **DNER** | 0.502533 | **MMP-10** | 0.43829 |
| **NT-3** | 0.493574 | **AXIN1** | 0.432081 |
| **IL6** | 0.492705 | **SIRT2** | 0.428004 |
| **CCL20** | 0.490269 | **CD6** | 0.410783 |
| **ST1A1** | 0.484046 | **CST5** | 0.403578 |
| **TRANCE** | 0.471819 | **DNER** | 0.403523 |
| **IL-17A** | 0.459138 | **CXCL5** | 0.393972 |
| **IL-12B** | 0.455406 | **TRANCE** | 0.38853 |
| **CDCP1** | 0.442235 | **4E-BP1** | 0.387824 |
| **Flt3L** | 0.441432 | **ST1A1** | 0.371915 |
| **FGF-23** | 0.437161 | **Flt3L** | 0.366895 |
| **TNFB** | 0.435949 | **TNFB** | 0.366326 |
| **IL-17C** | 0.43149 | **MMP-1** | 0.361266 |
| **CXCL9** | 0.425893 | **FGF-21** | 0.345594 |
| **IL7** | 0.416622 | **CCL20** | 0.323423 |
| **FGF-19** | 0.409555 | **IL-17C** | 0.323029 |
| **CST5** | 0.388284 | **IL-17A** | 0.322889 |
| **CCL25** | 0.380231 | **NT-3** | 0.29973 |
| **4E-BP1** | 0.36643 | **CCL25** | 0.295337 |
| **SCF** | 0.363786 | **IL-10RA** | 0.27711 |
| **FGF-21** | 0.348503 | **SCF** | 0.237503 |
| **MMP-1** | 0.336806 | **FGF-19** | 0.137192 |
| **IL-10RA** | 0.251743 |  |  |
|  |  |  |  |

# Supplemental Digital Content 5

**Replication cohort:** Medians and interquartile range for Groups 1, 2, and 3 for all 73 proteins. Data are expressed as normalized protein expression (NPX). Values of NPX are on a log2 scale. Furthest to the right we report P-values from the omnibus test (Kruskal Wallis test).

|  | **Group 1** | | |  | **Group 2** | | |  | **Group 3** | | |  |
| --- | --- | --- | --- | --- | --- | --- | --- | --- | --- | --- | --- | --- |
|  | 25th | 50th | 75th |  | 25th | 50th | 75th |  | 25th | 50th | 75th | **P-value** |
| 4E-BP1 | 7,54 | 8,15 | 8,46 |  | 8.03 | 8.77 | 9.23 |  | 7,74 | 8,34 | 9,18 | 7.90E-04 |
| ADA | 4,50 | 4,69 | 4,99 |  | 4.86 | 5.11 | 5.26 |  | 4,82 | 5,00 | 5,25 | 2.28E-07 |
| AXIN1 | 1,63 | 1,92 | 2,24 |  | 2.06 | 2.40 | 2.78 |  | 1,90 | 2,18 | 2,62 | 8.01E-05 |
| Beta-NGF | 1,91 | 2,02 | 2,17 |  | 2.39 | 2.50 | 2.83 |  | 2,10 | 2,24 | 2,36 | 1.50E-15 |
| CASP-8 | 2,43 | 2,64 | 2,87 |  | 2.90 | 3.08 | 3.41 |  | 2,70 | 2,89 | 3,08 | 4.47E-10 |
| CCL11 | 7,65 | 8,02 | 8,23 |  | 8.31 | 8.60 | 8.84 |  | 8,08 | 8,29 | 8,61 | 9.18E-10 |
| CCL19 | 9,35 | 9,61 | 10,04 |  | 10.23 | 10.94 | 11.33 |  | 9,81 | 10,13 | 10,71 | 1.63E-11 |
| CCL20 | 6,05 | 6,66 | 7,69 |  | 6.80 | 7.51 | 8.19 |  | 6,55 | 6,92 | 7,84 | 2.33E-03 |
| CCL23 | 9,66 | 9,93 | 10,11 |  | 10.22 | 10.52 | 10.93 |  | 10,09 | 10,26 | 10,47 | 2.96E-12 |
| CCL25 | 6,51 | 6,92 | 7,48 |  | 6.86 | 7.31 | 7.86 |  | 6,90 | 7,27 | 7,55 | 9.45E-03 |
| CCL28 | 2,65 | 2,97 | 3,16 |  | 3.16 | 3.47 | 3.71 |  | 2,99 | 3,26 | 3,46 | 5.34E-09 |
| CCL3 | 6,14 | 6,42 | 6,66 |  | 7.21 | 7.48 | 7.63 |  | 6,73 | 7,01 | 7,20 | 8.56E-21 |
| CCL4 | 6,50 | 6,78 | 7,20 |  | 7.49 | 7.77 | 8.12 |  | 6,99 | 7,26 | 7,61 | 5.25E-13 |
| CD244 | 6,81 | 7,02 | 7,22 |  | 7.31 | 7.47 | 7.72 |  | 7,23 | 7,41 | 7,53 | 1.42E-15 |
| CD40 | 11,47 | 11,66 | 11,83 |  | 12.21 | 12.40 | 12.81 |  | 11,88 | 12,07 | 12,20 | 2.56E-23 |
| CD5 | 4,70 | 4,92 | 5,18 |  | 5.44 | 5.57 | 5.80 |  | 5,18 | 5,30 | 5,52 | 3.07E-16 |
| CD6 | 5,53 | 5,82 | 6,10 |  | 5.86 | 6.08 | 6.54 |  | 5,84 | 6,05 | 6,34 | 4.81E-06 |
| CD8A | 9,76 | 10,11 | 10,41 |  | 10.39 | 10.78 | 11.07 |  | 9,95 | 10,41 | 10,91 | 2.61E-06 |
| CDCP1 | 4,16 | 4,46 | 4,83 |  | 4.92 | 5.44 | 5.60 |  | 4,61 | 4,85 | 5,25 | 1.29E-09 |
| CSF-1 | 10,54 | 10,68 | 10,83 |  | 11.15 | 11.24 | 11.37 |  | 10,86 | 10,95 | 11,07 | 1.56E-22 |
| CST5 | 6,27 | 6,48 | 6,80 |  | 6.73 | 7.01 | 7.35 |  | 6,53 | 6,88 | 7,19 | 1.24E-07 |
| CX3CL1 | 6,58 | 6,91 | 7,21 |  | 7.35 | 7.71 | 7.87 |  | 7,04 | 7,25 | 7,49 | 1.62E-12 |
| CXCL1 | 9,57 | 10,11 | 10,61 |  | 10.82 | 11.18 | 11.44 |  | 10,39 | 10,83 | 11,15 | 2.03E-09 |
| CXCL10 | 9,89 | 10,36 | 10,70 |  | 10.89 | 11.29 | 11.75 |  | 10,50 | 11,02 | 11,53 | 1.58E-11 |
| CXCL11 | 7,70 | 7,99 | 8,34 |  | 8.92 | 9.02 | 9.39 |  | 8,35 | 8,80 | 9,14 | 5.24E-17 |
| CXCL5 | 11,55 | 12,36 | 12,92 |  | 12.82 | 13.28 | 13.52 |  | 12,21 | 12,89 | 13,22 | 1.18E-05 |
| CXCL6 | 8,77 | 9,09 | 9,39 |  | 9.76 | 10.12 | 10.63 |  | 9,28 | 9,64 | 10,06 | 5.77E-14 |
| CXCL9 | 7,54 | 8,00 | 8,35 |  | 8.56 | 9.12 | 9.42 |  | 7,99 | 8,50 | 9,03 | 4.58E-10 |
| DNER | 9,14 | 9,36 | 9,55 |  | 9.40 | 9.57 | 9.77 |  | 9,35 | 9,55 | 9,74 | 5.91E-05 |
| EN-RAGE | 4,79 | 5,24 | 5,79 |  | 5.58 | 6.18 | 6.49 |  | 5,03 | 5,45 | 6,09 | 1.12E-04 |
| FGF-19 | 7,71 | 8,24 | 8,79 |  | 7.92 | 8.61 | 9.01 |  | 7,89 | 8,44 | 8,99 | 1.64E-01 |
| FGF-21 | 5,07 | 5,85 | 6,70 |  | 6.59 | 7.20 | 8.26 |  | 5,32 | 6,14 | 6,76 | 5.04E-06 |
| Flt3L | 9,53 | 9,76 | 9,91 |  | 9.83 | 10.06 | 10.37 |  | 9,70 | 9,94 | 10,22 | 4.57E-06 |
| GDNF | 2,67 | 2,85 | 3,05 |  | 3.27 | 3.50 | 3.62 |  | 2,90 | 3,11 | 3,30 | 1.14E-11 |
| HGF | 9,36 | 9,67 | 9,83 |  | 10.34 | 10.52 | 10.81 |  | 9,73 | 9,98 | 10,19 | 1.81E-18 |
| IL10 | 4,00 | 4,23 | 4,46 |  | 4.60 | 4.92 | 5.42 |  | 4,31 | 4,47 | 4,75 | 7.60E-11 |
| IL-10RA | 0,99 | 1,13 | 1,42 |  | 1.17 | 1.30 | 1.46 |  | 1,09 | 1,20 | 1,47 | 8.78E-03 |
| IL-10RB | 6,28 | 6,51 | 6,68 |  | 6.92 | 7.14 | 7.27 |  | 6,64 | 6,82 | 6,96 | 2.79E-17 |
| IL-12B | 6,16 | 6,70 | 7,00 |  | 7.20 | 7.44 | 7.85 |  | 6,81 | 7,18 | 7,55 | 8.31E-11 |
| IL-15RA | 1,19 | 1,39 | 1,51 |  | 1.79 | 1.88 | 1.98 |  | 1,52 | 1,60 | 1,79 | 2.40E-19 |
| IL-17A | 1,89 | 2,16 | 2,50 |  | 2.40 | 2.61 | 3.70 |  | 2,21 | 2,53 | 2,82 | 2.75E-05 |
| IL-17C | 2,24 | 2,59 | 3,03 |  | 2.76 | 3.17 | 3.80 |  | 2,54 | 2,84 | 3,30 | 1.67E-04 |
| IL18 | 8,18 | 8,43 | 8,82 |  | 8.74 | 9.11 | 9.65 |  | 8,59 | 8,85 | 9,26 | 7.66E-09 |
| IL-18R1 | 8,50 | 8,79 | 9,05 |  | 9.37 | 9.69 | 9.78 |  | 8,97 | 9,13 | 9,46 | 1.86E-15 |
| IL6 | 3,78 | 4,14 | 4,49 |  | 4.81 | 5.31 | 6.13 |  | 4,15 | 4,47 | 4,87 | 1.23E-10 |
| IL7 | 4,28 | 4,68 | 5,05 |  | 5.10 | 5.43 | 5.79 |  | 4,64 | 4,91 | 5,36 | 6.64E-09 |
| IL8 | 7,01 | 7,25 | 7,62 |  | 7.80 | 8.16 | 8.80 |  | 7,35 | 7,75 | 8,11 | 1.32E-11 |
| LAPTGF-beta-1 | 7,72 | 7,93 | 8,26 |  | 8.44 | 8.61 | 8.82 |  | 8,12 | 8,47 | 8,63 | 2.53E-15 |
| LIF-R | 4,19 | 4,32 | 4,47 |  | 4.71 | 4.90 | 5.18 |  | 4,44 | 4,59 | 4,71 | 2.12E-18 |
| MCP-1 | 11,54 | 11,82 | 12,08 |  | 12.38 | 12.56 | 12.72 |  | 12,05 | 12,26 | 12,48 | 1.02E-15 |
| MCP-2 | 9,48 | 9,88 | 10,33 |  | 10.41 | 10.61 | 11.06 |  | 9,95 | 10,40 | 10,74 | 6.40E-10 |
| MCP-3 | 2,31 | 2,60 | 2,82 |  | 3.01 | 3.40 | 3.82 |  | 2,68 | 2,89 | 3,28 | 1.46E-12 |
| MCP-4 | 14,01 | 14,44 | 14,88 |  | 15.06 | 15.41 | 15.71 |  | 14,66 | 15,06 | 15,53 | 2.76E-11 |
| MMP-1 | 10,61 | 11,69 | 12,51 |  | 11.99 | 12.77 | 13.35 |  | 11,21 | 12,06 | 12,75 | 1.32E-04 |
| MMP-10 | 6,65 | 7,01 | 7,44 |  | 7.35 | 7.72 | 8.12 |  | 7,06 | 7,39 | 7,62 | 1.10E-07 |
| NT-3 | 2,28 | 2,41 | 2,61 |  | 2.58 | 2.83 | 3.10 |  | 2,33 | 2,50 | 2,74 | 6.36E-06 |
| OPG | 10,15 | 10,39 | 10,63 |  | 10.78 | 10.98 | 11.23 |  | 10,47 | 10,66 | 10,89 | 1.71E-12 |
| OSM | 4,97 | 5,59 | 6,23 |  | 6.47 | 6.90 | 7.04 |  | 5,32 | 5,94 | 6,49 | 2.25E-09 |
| PD-L1 | 6,73 | 6,98 | 7,20 |  | 7.61 | 7.72 | 7.93 |  | 7,24 | 7,39 | 7,55 | 1.32E-21 |
| SCF | 9,74 | 10,00 | 10,19 |  | 9.95 | 10.17 | 10.42 |  | 9,90 | 10,06 | 10,35 | 8.07E-03 |
| SIRT2 | 3,26 | 3,66 | 4,17 |  | 3.74 | 4.54 | 4.77 |  | 3,55 | 3,98 | 4,65 | 1.02E-04 |
| SLAMF1 | 3,47 | 3,73 | 3,99 |  | 4.28 | 4.61 | 5.01 |  | 3,96 | 4,14 | 4,48 | 1.27E-12 |
| ST1A1 | 1,66 | 1,96 | 2,45 |  | 2.12 | 2.41 | 2.86 |  | 1,94 | 2,32 | 2,70 | 4.85E-04 |
| STAMBP | 4,43 | 4,72 | 5,11 |  | 4.91 | 5.31 | 5.65 |  | 4,66 | 4,94 | 5,46 | 2.58E-05 |
| TGF-alpha | 4,79 | 5,06 | 5,43 |  | 5.83 | 6.24 | 6.59 |  | 5,13 | 5,41 | 5,86 | 3.87E-13 |
| TNFB | 4,93 | 5,17 | 5,41 |  | 5.34 | 5.46 | 5.75 |  | 5,18 | 5,50 | 5,68 | 1.10E-06 |
| TNFRSF9 | 6,85 | 7,16 | 7,50 |  | 7.95 | 8.17 | 8.37 |  | 7,44 | 7,73 | 7,95 | 4.01E-20 |
| TNFSF14 | 3,94 | 4,30 | 4,71 |  | 5.05 | 5.41 | 5.91 |  | 4,18 | 4,71 | 5,06 | 4.89E-12 |
| TRAIL | 8,57 | 8,75 | 8,95 |  | 9.07 | 9.24 | 9.34 |  | 8,86 | 9,03 | 9,23 | 2.99E-12 |
| TRANCE | 4,43 | 4,95 | 5,34 |  | 5.25 | 5.59 | 5.94 |  | 4,98 | 5,38 | 5,67 | 8.09E-06 |
| TWEAK | 9,71 | 10,07 | 10,27 |  | 10.21 | 10.37 | 10.68 |  | 10,09 | 10,29 | 10,50 | 9.68E-08 |
| uPA | 9,41 | 9,67 | 9,82 |  | 9.97 | 10.12 | 10.29 |  | 9,73 | 9,93 | 10,11 | 5.84E-13 |
| VEGFA | 10,30 | 10,63 | 10,95 |  | 11.22 | 11.62 | 11.80 |  | 10,66 | 11,05 | 11,26 | 4.04E-13 |
|  |  |  |  |  |  |  |  |  |  |  |  |  |

# Supplemental Digital Content 6

Please insert link here to downloaded Excel file.

# Supplemental Digital Content 7

**Clinical data in the 3 groups defined by hierarchical cluster analysis (HCA) (Exploratory and replication cohorts together).**

**Contents**

[Age 14](#_Toc73347391)

[Body Mass Index (BMI) 15](#_Toc73347392)

[HbA1c% 16](#_Toc73347393)

[Toronto Clinical Scoring System (TCSS) 17](#_Toc73347394)

[Douleur Neuropathique en 4 Questions (DN4) 18](#_Toc73347395)

[Brief Pain Inventory – worst (BPI_worst) 19](#_Toc73347396)

[Brief Pain Inventory – least (BPI_least) 20](#_Toc73347397)

[Brief Pain Inventory – average (BPI_average) 21](#_Toc73347398)

[Brief Pain Inventory – now (BPI_now) 22](#_Toc73347399)

[BPI_pain severity subscore 23](#_Toc73347400)

[NPSI_Superficial_Spontaneous 24](#_Toc73347401)

[NPSI_Deep_Spontaneous 25](#_Toc73347402)

[NPSI_Paroxysmal 26](#_Toc73347403)

[NPSI_Evoked 27](#_Toc73347404)

[NPSI_Paraesthesia 28](#_Toc73347405)

[NPSI_Total 29](#_Toc73347406)

[PainDETECT 30](#_Toc73347407)

[PCS_Rumination 31](#_Toc73347408)

[PCS_Magnification 32](#_Toc73347409)

[PCS_Helplessness 33](#_Toc73347410)

[PCS_Total 34](#_Toc73347411)

## Age


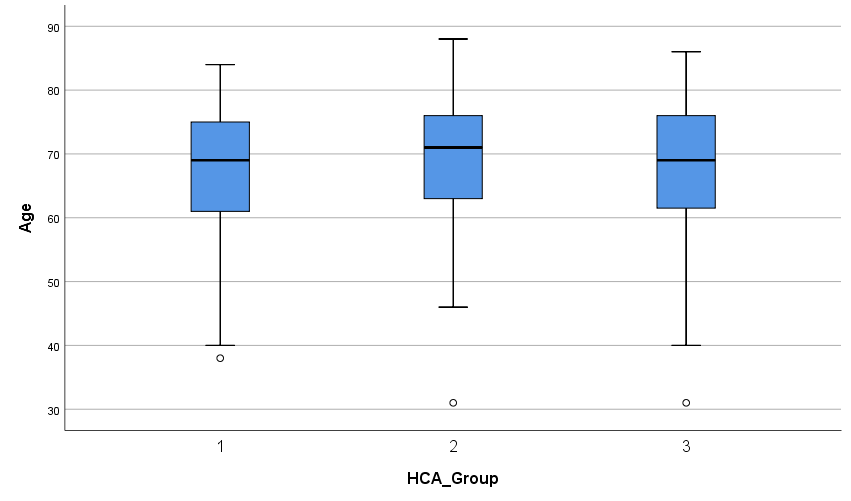


## Body Mass Index (BMI)


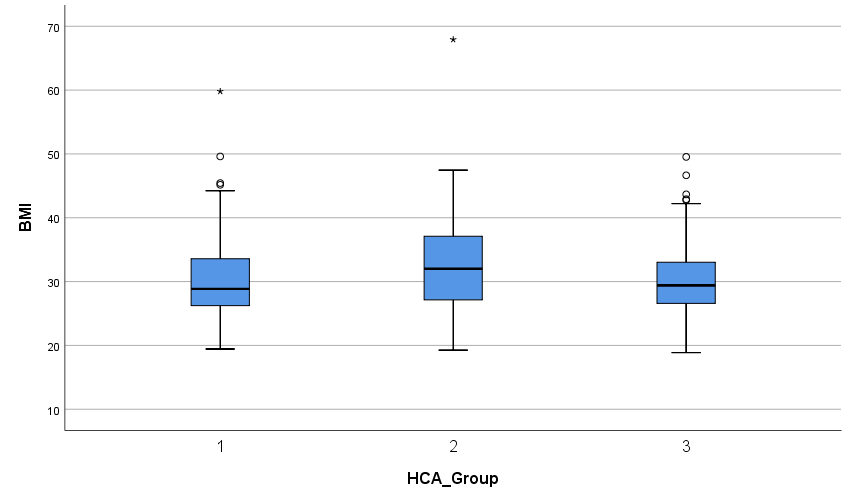


## HbA1c%


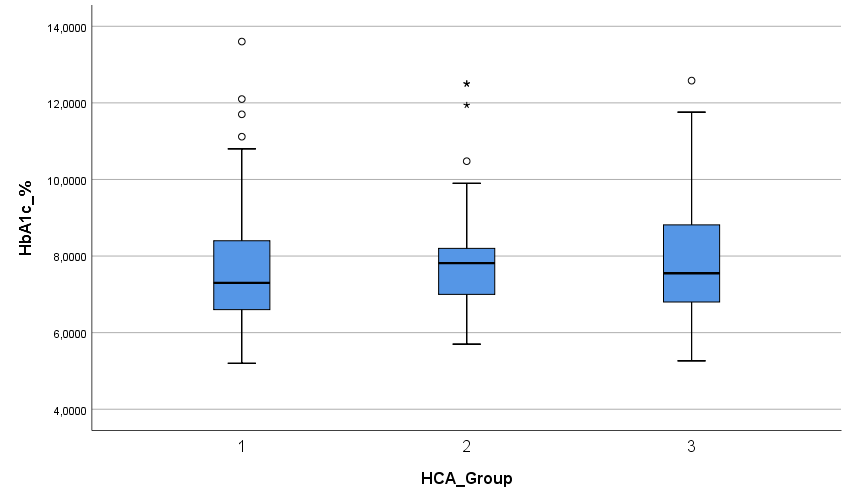


## Toronto Clinical Scoring System (TCSS)


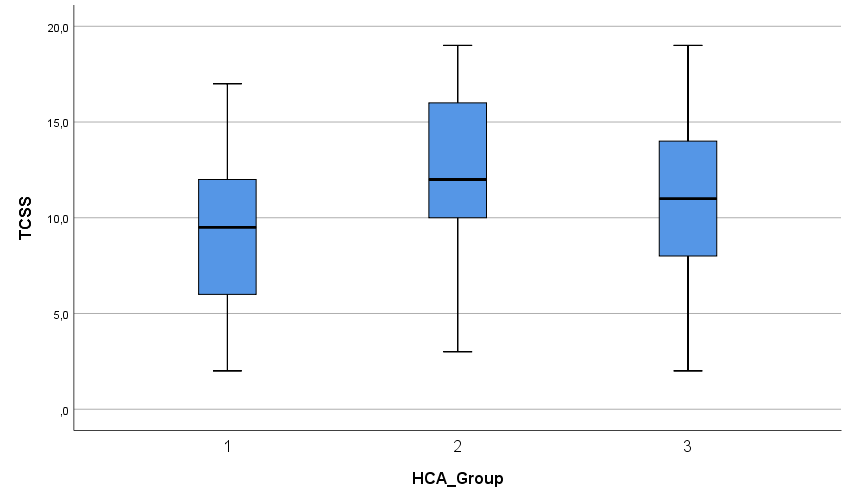


## Douleur Neuropathique en 4 Questions (DN4)


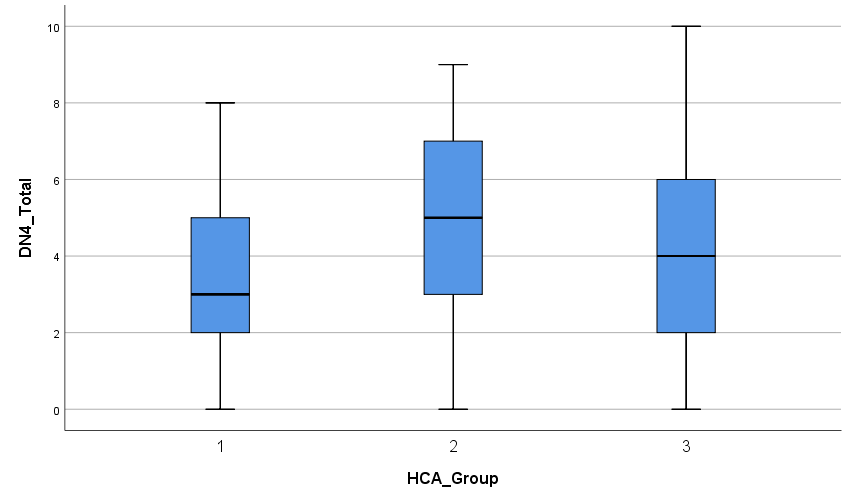


## Brief Pain Inventory – worst (BPI_worst)


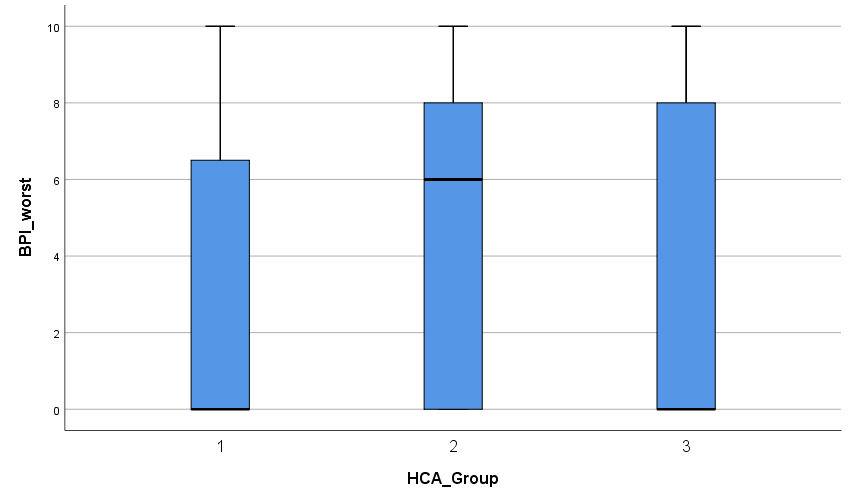


## Brief Pain Inventory – least (BPI_least)


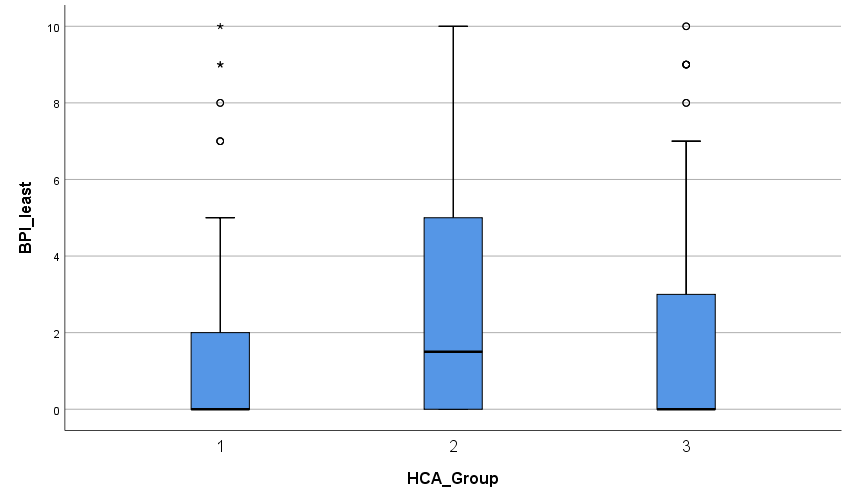


## Brief Pain Inventory – average (BPI_average)


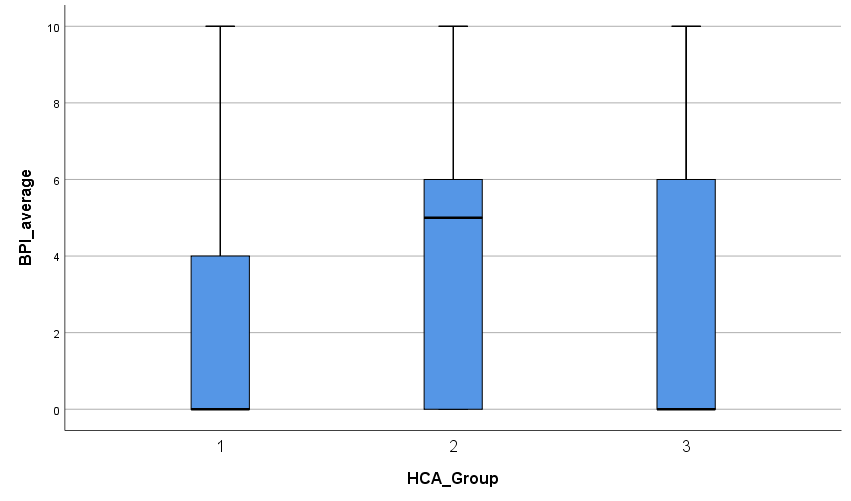


## Brief Pain Inventory – now (BPI_now)


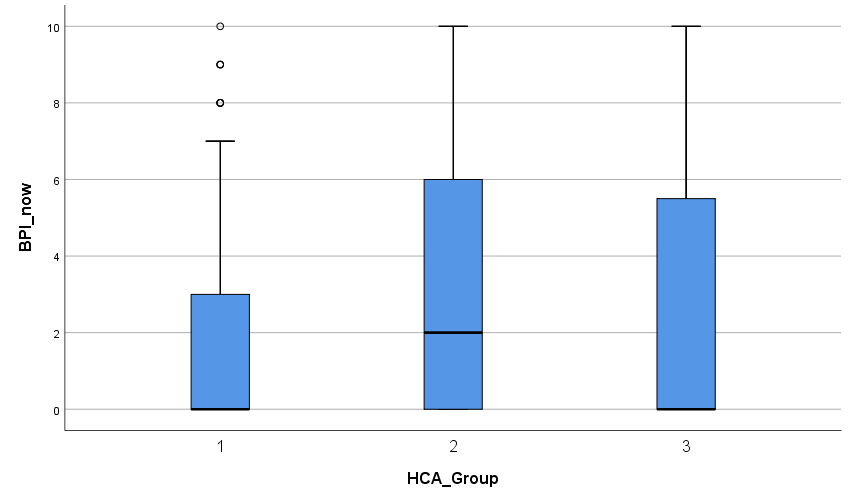


## BPI_pain severity subscore


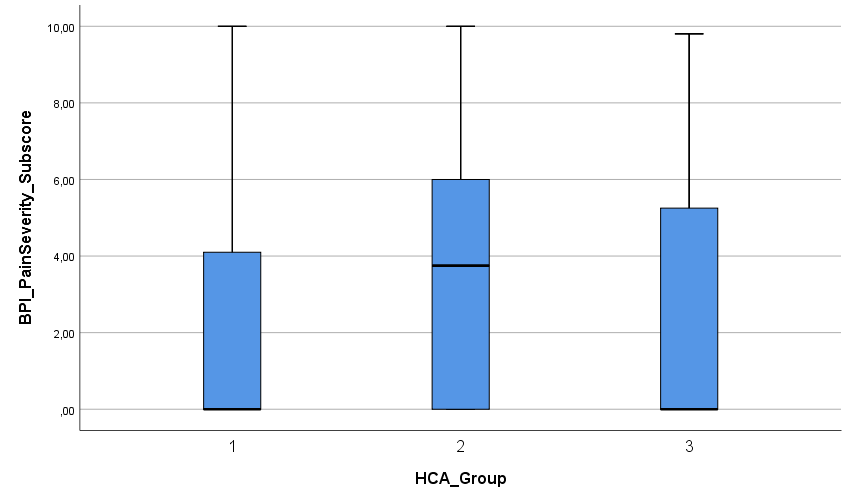


## NPSI_Superficial_Spontaneous


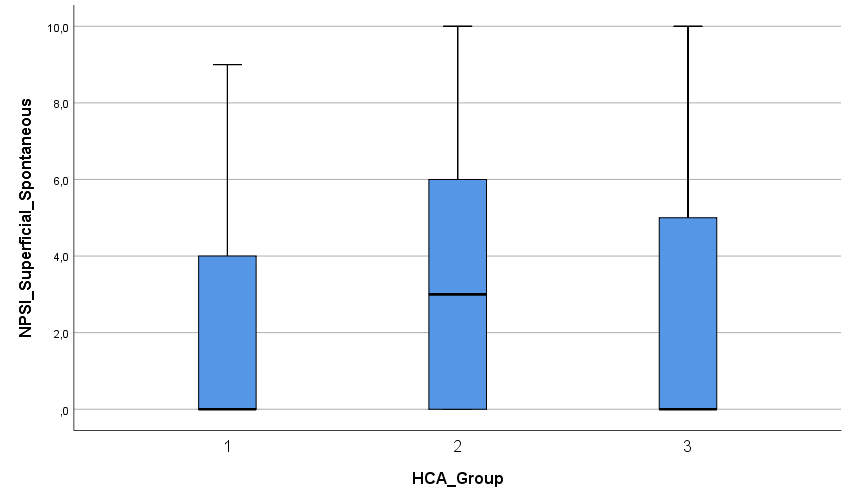


Note: NPSI = Neuropathic Pain Symptom Inventory

## NPSI_Deep_Spontaneous


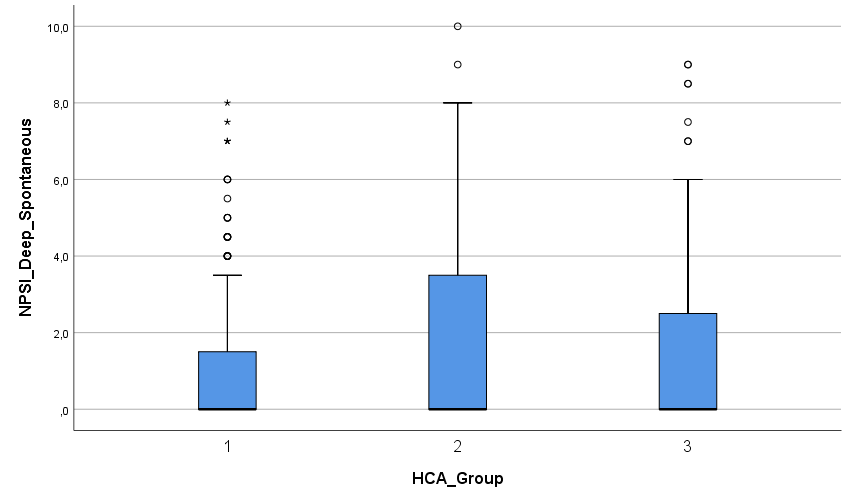


Note: NPSI = Neuropathic Pain Symptom Inventory

## NPSI_Paroxysmal


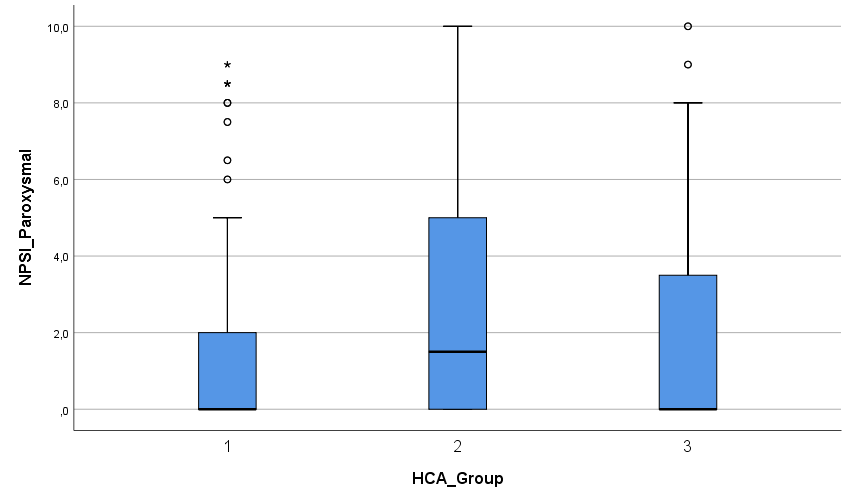


Note: NPSI = Neuropathic Pain Symptom Inventory

## NPSI_Evoked


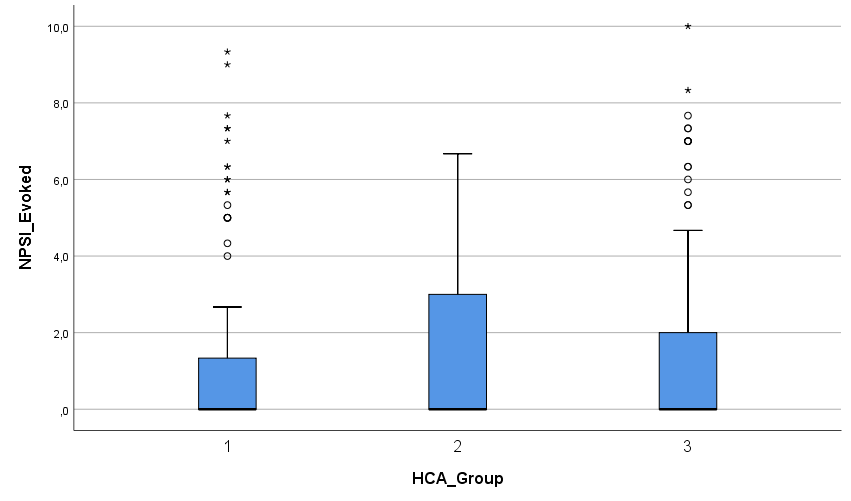


Note: NPSI = Neuropathic Pain Symptom Inventory

## NPSI_Paraesthesia


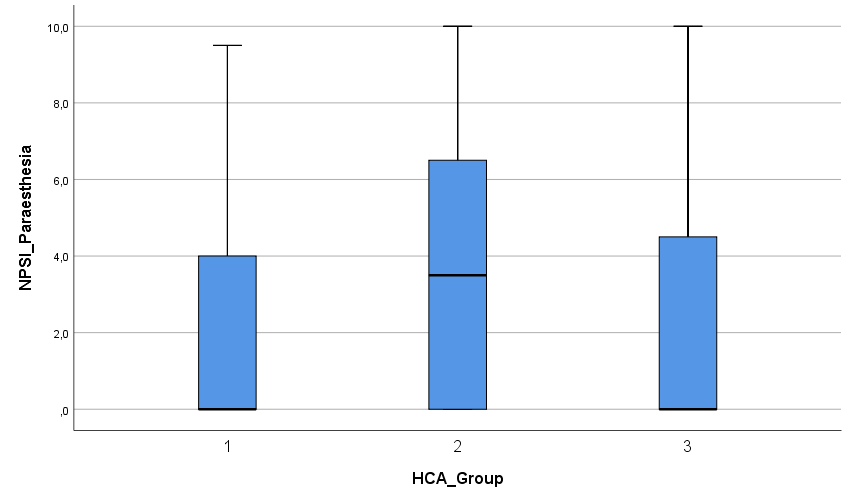


Note: NPSI = Neuropathic Pain Symptom Inventory

## NPSI_Total


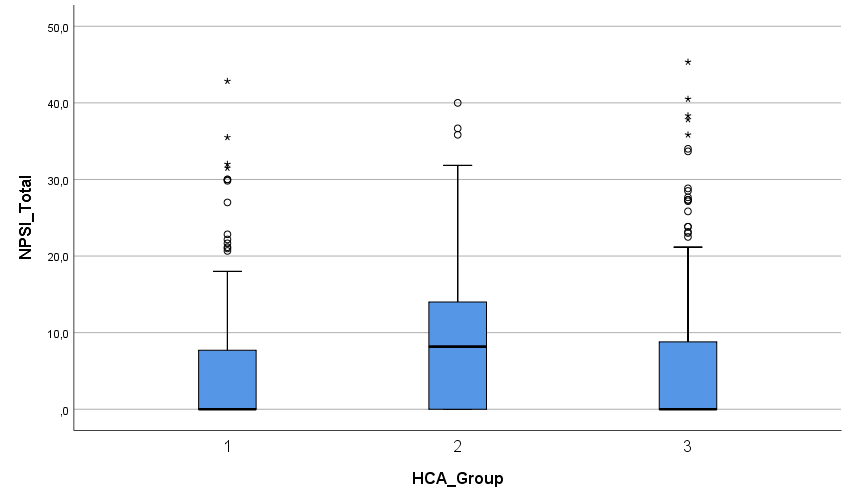


Note: NPSI = Neuropathic Pain Symptom Inventory

## PainDETECT


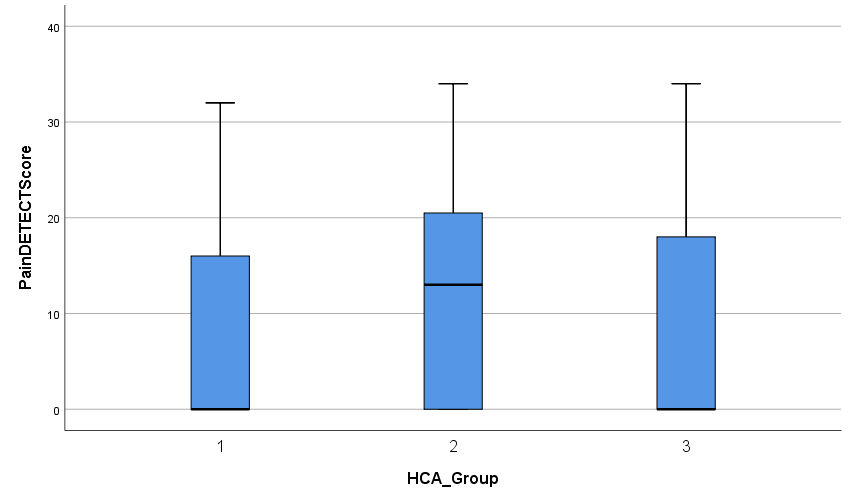


## PCS_Rumination


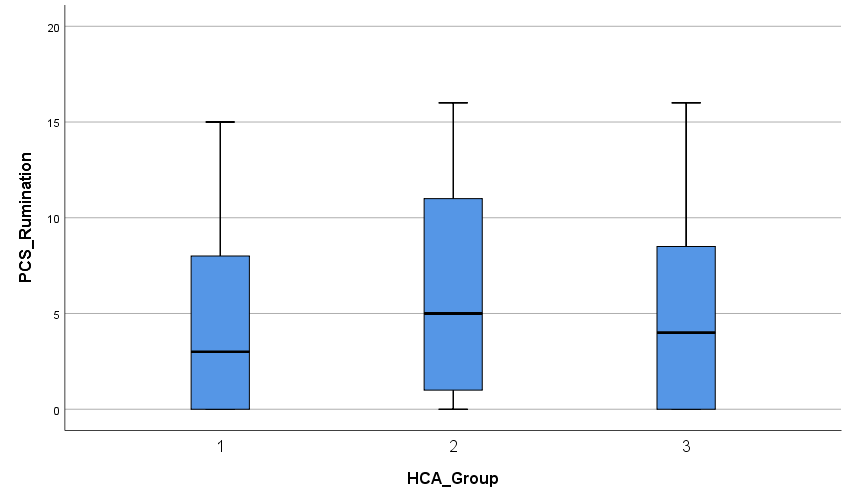


Note: PCS = Pain Catastrophizing Scale

## PCS_Magnification


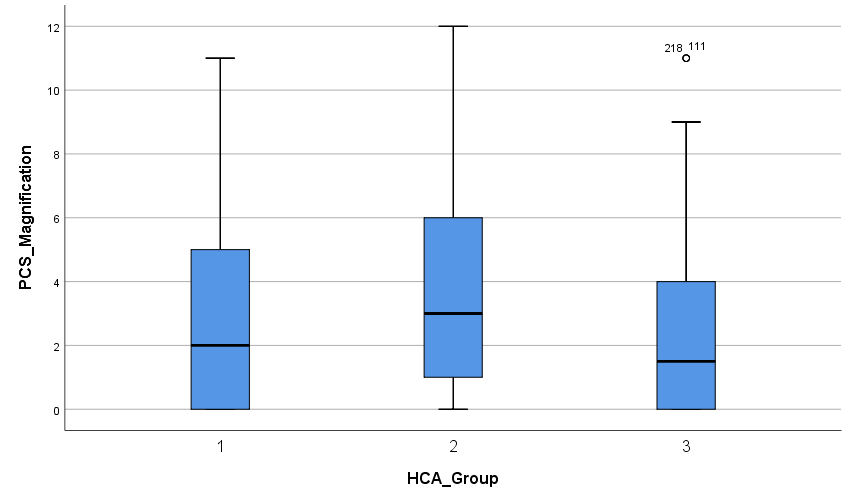


Note: PCS = Pain Catastrophizing Scale

## PCS_Helplessness


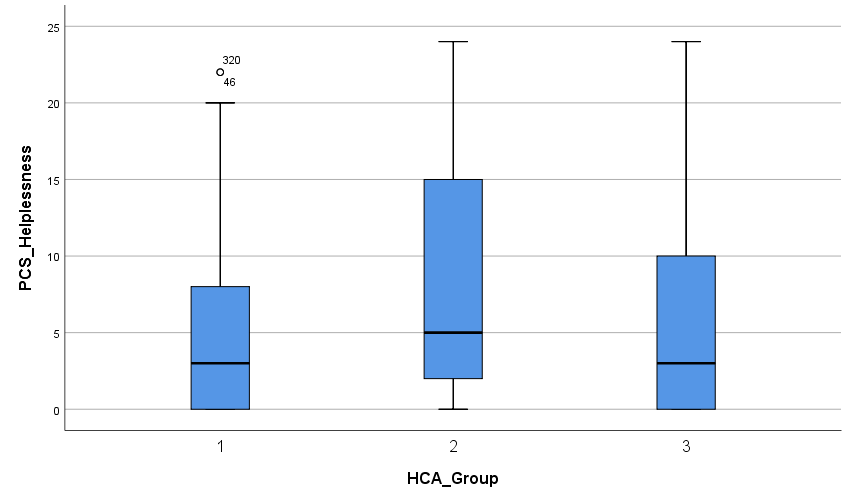


Note: PCS = Pain Catastrophizing Scale

## PCS_Total


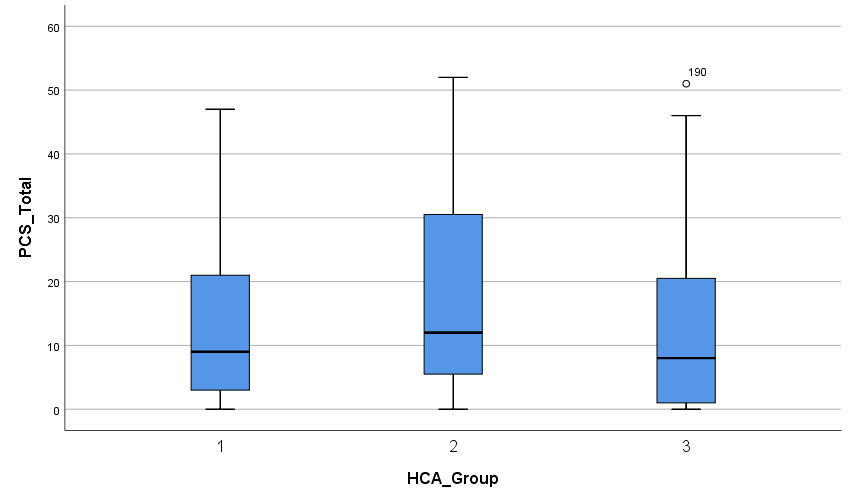


Note: PCS = Pain Catastrophizing Scale

# Supplemental Digital Content 8

## Painful vs. painless DPN (exploratory cohort)

n=179, 1 latent variable, R^2^=0.05, Q^2^=0.01, CV-ANOVA p=0.51

The following table shows the top 20 proteins of the model, the negative sign indicating higher levels in painful DPN. Notably, 16 of the top 20 proteins according to Table 3 (exploratory cohort) are present here. Hence, although the model is not significant and has low R^2^ and Q^2^, there are still strong similiarities to our main results.

| **Proteins** | **p(corr)** |
| --- | --- |
| **HGF** | -0.84 |
| **CSF-1** | -0.82 |
| **CASP-8** | -0.77 |
| **MCP-1** | -0.76 |
| **TGF-alpha** | -0.75 |
| **TNFSF14** | -0.75 |
| **CD40** | -0.74 |
| **EN-RAGE** | -0.74 |
| **LAP TGF-beta-1** | -0.72 |
| **CD5** | -0.71 |
| **OSM** | -0.70 |
| **CD244** | -0.70 |
| **CXCL6** | -0.69 |
| **CD6** | -0.69 |
| **LIF-R** | -0.68 |
| **IL-10RB** | -0.66 |
| **CXCL11** | -0.66 |
| **PD-L1** | -0.65 |
| **STAMPB** | -0.65 |
| **SIRT2** | -0.63 |

## Patients with moderate/severe pain vs. painless patients (exploratory cohort)

OPLS-DA, n=142, 1 latent variable, R^2^=0.07, Q^2^=0.02, CV-ANOVA p=0.18

The following table shows the top 20 proteins of the model. Notably, 17 of the top 20 proteins according to Table 3 (exploratory cohort) are present here. Hence, although the model is not significant and has low R^2^ and Q^2^, there are still strong similiarities to our main results.

| **Proteins** | **p(corr)** |
| --- | --- |
| **HGF** | -0.84 |
| **CSF-1** | -0.81 |
| **MCP-1** | -0.79 |
| **CASP-8** | -0.78 |
| **TGF-alpha** | -0.76 |
| **CD6** | -0.75 |
| **EN-RAGE** | -0.75 |
| **LAP TGF-beta-1** | -0.74 |
| **TNFSF14** | -0.74 |
| **CCL3** | -0.74 |
| **CD244** | -0.73 |
| **CD5** | -0.72 |
| **CD40** | -0.72 |
| **CXCL6** | -0.71 |
| **LIF-R** | -0.70 |
| **OSM** | -0.69 |
| **CCL4** | -0.68 |
| **IL-10RB** | -0.66 |
| **CXCL11** | -0.65 |
| **TRAIL** | -0.64 |

# Supplemental Digital Content 9

UniProt and PubMed were accessed 30 June 2021.

## Hepatocyte growth factor (HGF)

- **Function according to UniProt** (P14210): “Potent mitogen for mature parenchymal hepatocyte cells, seems to be a hepatotrophic factor, and acts as a growth factor for a broad spectrum of tissues and cell types. Activating ligand for the receptor tyrosine kinase MET by binding to it and promoting its dimerization.”
- **PubMed search ("Hepatocyte growth factor") AND (pain):** 100 hits, and titles with publication date 2011-2021 (64 hits) were scanned for relevant papers.
- **Selected neuropathic pain-related aspects according to the PubMed search:**
  - HGF is increased in injured peripheral nerves, and HGF seems to play a role in Schwann cell-mediated nerve repair [1].
  - HGF gene therapy has been proposed as a possible treatment for diabetic neuropathy and neuropathic pain [2-5].
  - There are potentially relevant animal models mentioning HGF [6, 7].

## Macrophage colony-stimulating factor 1 (CSF-1)

- **Function according to UniProt** (P09603): “Cytokine that plays an essential role in the regulation of survival, proliferation and differentiation of hematopoietic precursor cells, especially mononuclear phagocytes, such as macrophages and monocytes. Promotes the release of proinflammatory chemokines, and thereby plays an important role in innate immunity and in inflammatory processes. Plays an important role in the regulation of osteoclast proliferation and differentiation, the regulation of bone resorption, and is required for normal bone development. Required for normal male and female fertility. Promotes reorganization of the actin cytoskeleton, regulates formation of membrane ruffles, cell adhesion and cell migration. Plays a role in lipoprotein clearance.”
- **PubMed search ("Macrophage colony-stimulating factor 1" OR CSF-1) AND (pain):** 344 hits, and titles with publication date 2018-2021 (63 hits) were scanned for relevant papers.
- **Selected neuropathic pain-related aspects according to the PubMed search:**
  - There is a recent review from 2021 in *Pain Reports* concerning CSF-1 and neuropathic pain [8].
  - The **CSF-1**/CSF-1R/DAP12 pathway mediates neuron-microglia communication in the dorsal horn, as do the P2X4/BDNF/GABAA and P2X7/Cathepsin S/Fractalkine/CX3CR1 pathways [9, 10].
  - There are other potentially relevant animal models mentioning CSF-1 [6, 11-19].

## CD40L receptor (CD40)

- **Function according to UniProt ID** (P25942): “Receptor for TNFSF5/CD40LG (PubMed:[31331973](https://www.uniprot.org/citations/31331973)).Transduces TRAF6- and MAP3K8-mediated signals that activate ERK in macrophages and B cells, leading to induction of immunoglobulin secretion (By similarity).”
- **PubMed search ("CD40L receptor" OR CD40) AND (pain):** 140 hits, and titles with publication date 2011-2021 (59 hits) were scanned for relevant papers.
- **Selected neuropathic pain-related aspects according to the PubMed search:**
  - CD40, a member of the TNF-receptor superfamily, has an increased expression in diabetic nerves and seems to be a key molecule for the upregulation of hypoxia-inducible factor-1α (HIF-1α) [20]. A relationship between HIF-1α (a regulator of oxygen homeostasis) and pain intensity in polyneuropathy patients has been reported [21].
  - There are potentially relevant animal models mentioning CD40 [22-29].

## References

1. Ko KR, Lee J, Lee D, et al. Hepatocyte Growth Factor (HGF) Promotes Peripheral Nerve Regeneration by Activating Repair Schwann Cells. Sci Rep. 2018;8:8316.

2. Kessler JA, Smith AG, Cha BS, et al. Double-blind, placebo-controlled study of HGF gene therapy in diabetic neuropathy. Ann Clin Transl Neurol. 2015;2:465-78.

3. Kessler JA, Shaibani A, Sang CN, et al. Gene therapy for diabetic peripheral neuropathy: A randomized, placebo-controlled phase III study of VM202, a plasmid DNA encoding human hepatocyte growth factor. Clin Transl Sci. 2021;14:1176-84.

4. Hu C, Lu Y, Cheng X, et al. Gene therapy for neuropathic pain induced by spared nerve injury with naked plasmid encoding hepatocyte growth factor. J Gene Med. 2017;19.

5. Ajroud-Driss S, Christiansen M, Allen JA, et al. Phase 1/2 open-label dose-escalation study of plasmid DNA expressing two isoforms of hepatocyte growth factor in patients with painful diabetic peripheral neuropathy. Mol Ther. 2013;21:1279-86.

6. Nho B, Lee J, Lee J, et al. Effective control of neuropathic pain by transient expression of hepatocyte growth factor in a mouse chronic constriction injury model. Faseb j. 2018;32:5119-31.

7. Murakami K, Kuniyoshi K, Iwakura N, et al. Vein wrapping for chronic nerve constriction injury in a rat model: study showing increases in VEGF and HGF production and prevention of pain-associated behaviors and nerve damage. The Journal of bone and joint surgery. American volume. 2014;96:859-67.

8. Yu X, Basbaum A,Guan Z. Contribution of colony-stimulating factor 1 to neuropathic pain. Pain Rep. 2021;6:e883.

9. Malcangio M. Role of the immune system in neuropathic pain. Scand J Pain. 2019;20:33-7.

10. Tozaki-Saitoh H,Tsuda M. Microglia-neuron interactions in the models of neuropathic pain. Biochem Pharmacol. 2019;169:113614.

11. Lee J, Hwang H,Lee SJ. Distinct roles of GT1b and CSF-1 in microglia activation in nerve injury-induced neuropathic pain. Molecular pain. 2021;17:17448069211020918.

12. Yang G, Tan Q, Li Z, et al. The AMPK pathway triggers autophagy during CSF1-induced microglial activation and may be implicated in inducing neuropathic pain. J Neuroimmunol. 2020;345:577261.

13. Yu X, Liu H, Hamel KA, et al. Dorsal root ganglion macrophages contribute to both the initiation and persistence of neuropathic pain. Nat Commun. 2020;11:264.

14. Liu L, Xu D, Wang T, et al. Epigenetic reduction of miR-214-3p upregulates astrocytic colony-stimulating factor-1 and contributes to neuropathic pain induced by nerve injury. Pain. 2020;161:96-108.

15. Zhou LJ, Peng J, Xu YN, et al. Microglia Are Indispensable for Synaptic Plasticity in the Spinal Dorsal Horn and Chronic Pain. Cell reports. 2019;27:3844-59.e6.

16. Boakye PA, Rancic V, Whitlock KH, et al. Receptor dependence of BDNF actions in superficial dorsal horn: relation to central sensitization and actions of macrophage colony stimulating factor 1. J Neurophysiol. 2019;121:2308-22.

17. Nicol LSC, Thornton P, Hatcher JP, et al. Central inhibition of granulocyte-macrophage colony-stimulating factor is analgesic in experimental neuropathic pain. Pain. 2018;159:550-9.

18. Yang G, Chen L, Gao Z, et al. Implication of microglia activation and CSF-1/CSF-1Rpathway in lumbar disc degeneration-related back pain. Molecular pain. 2018;14:1744806918811238.

19. Lee S, Shi XQ, Fan A, et al. Targeting macrophage and microglia activation with colony stimulating factor 1 receptor inhibitor is an effective strategy to treat injury-triggered neuropathic pain. Molecular pain. 2018;14:1744806918764979.

20. Kan HW, Hsieh JH, Chien HF, et al. CD40-mediated HIF-1α expression underlying microangiopathy in diabetic nerve pathology. Dis Model Mech. 2018;11.

21. Sloan G, Shillo P, Selvarajah D, et al. A new look at painful diabetic neuropathy. Diabetes Res Clin Pract. 2018;144:177-91.

22. Cao L,Malon JT. Anti-nociceptive Role of CXCL1 in a Murine Model of Peripheral Nerve Injury-induced Neuropathic Pain. Neuroscience. 2018;372:225-36.

23. Kwiatkowski K, Piotrowska A, Rojewska E, et al. The RS504393 Influences the Level of Nociceptive Factors and Enhances Opioid Analgesic Potency in Neuropathic Rats. Journal of neuroimmune pharmacology : the official journal of the Society on NeuroImmune Pharmacology. 2017;12:402-19.

24. Jurga AM, Rojewska E, Piotrowska A, et al. Blockade of Toll-Like Receptors (TLR2, TLR4) Attenuates Pain and Potentiates Buprenorphine Analgesia in a Rat Neuropathic Pain Model. Neural Plast. 2016;2016:5238730.

25. Rojewska E, Korostynski M, Przewlocki R, et al. Expression profiling of genes modulated by minocycline in a rat model of neuropathic pain. Molecular pain. 2014;10:47.

26. Draleau K, Maddula S, Slaiby A, et al. Phenotypic Identification of Spinal Cord-Infiltrating CD4(+) T Lymphocytes in a Murine Model of Neuropathic Pain. J Pain Relief. 2014;Suppl 3:003.

27. Cao L, Beaulac H,Eurich A. Differential lumbar spinal cord responses among wild type, CD4 knockout, and CD40 knockout mice in spinal nerve L5 transection-induced neuropathic pain. Molecular pain. 2012;8:88.

28. Malon JT, Maddula S, Bell H, et al. Involvement of calcitonin gene-related peptide and CCL2 production in CD40-mediated behavioral hypersensitivity in a model of neuropathic pain. Neuron Glia Biol. 2011;7:117-28.

29. Grace PM, Rolan PE,Hutchinson MR. Peripheral immune contributions to the maintenance of central glial activation underlying neuropathic pain. Brain, behavior, and immunity. 2011;25:1322-32.

# Supplemental Digital Content 10

Using the 14 proteins as per section 3.3, we conducted a network analysis. Hence, this resulted in a highly significant enriched network (**Fig. 5**). This analysis clearly indicated that most of these proteins form a protein-protein network according to existing bioinformatic data. The network analysis underscores the need in future research to consider that proteins work in networks instead of as a starting point focus upon one or a few candidate proteins. The KEGG pathway analysis indicated that the proteins were involved in cytokine-cytokine receptor interaction but also gave support for involvement of pathways known to be involved in pain such as Ras-Raf-MEK-ERK (development and maintenance of neuropathic pain) [2; 5], signaling of the proinflammatory IL-17 [3] and TNF [1] and PI3K-Akt signaling (important role in pain sensitivity) [4]. In agreement with the KEGG analysis, important Biological processes were responses, pathways and receptor binding relating to cytokines. But also, regulation of immune system, regulation of intracellular signal transduction and regulation of cells (activation, migration, adhesion) were biological process terms with low FDR. The terms of molecular function reflected cytokine and growth factor receptor binding/ activity. It was reasonable that cellular component identified terms related to extracellular aspects since the samples were from serum. Taken together, the bioinformatic analysis indicated involvement of complex mainly cytokine related mechanisms in Group 2 in comparison to Group (1+3).

**References**

[1] Andrade P, Visser-Vandewalle V, Hoffmann C, Steinbusch HW, Daemen MA, Hoogland G. Role of TNF-alpha during central sensitization in preclinical studies. Neurol Sci 2011;32(5):757-771.

[2] Hu C, Zhao YT, Cui YB, Zhang HH, Huang GL, Liu Y, Liu YF. Wnt/beta-Catenin Signaling Contributes to Vincristine-Induced Neuropathic Pain. Physiol Res 2020;69(4):701-710.

[3] Luo H, Liu HZ, Zhang WW, Matsuda M, Lv N, Chen G, Xu ZZ, Zhang YQ. Interleukin-17 Regulates Neuron-Glial Communications, Synaptic Transmission, and Neuropathic Pain after Chemotherapy. Cell Rep 2019;29(8):2384-2397 e2385.

[4] Zhang M, Jin F, Zhu Y, Qi F. Peripheral FGFR1 Regulates Myofascial Pain in Rats via the PI3K/AKT Pathway. Neuroscience 2020;436:1-10.

[5] Zhang YY, Song N, Liu F, Lin J, Liu MK, Huang CL, Liao DQ, Zhou C, Wang H, Shen JF. Activation of the RAS/B-RAF-MEK-ERK pathway in satellite glial cells contributes to substance p-mediated orofacial pain. Eur J Neurosci 2020;51(11):2205-2218.
